# Integrative Multiomics Profiling of Mouse Hippocampus Reveals Transcriptional Upregulation of Interferon‐Stimulated Genes Through PU.1 Regulator in Microglial Activation Induced by Chronic Cerebral Hypoperfusion

**DOI:** 10.1002/mco2.70157

**Published:** 2025-04-15

**Authors:** Zengyu Zhang, Dewen Ru, Zhuohang Liu, Zimin Guo, Lei Zhu, Yuan Zhang, Min Chu, Yong Wang, Jing Zhao

**Affiliations:** ^1^ Department of Neurology Minhang Hospital Fudan University Shanghai China; ^2^ Shanghai Medical College Fudan University Shanghai China; ^3^ Department of Neurosurgery Jinshan Hospital Fudan University Shanghai China; ^4^ Department of Neurosurgery Huashan Hospital Fudan University Shanghai China; ^5^ Department of Neurology Shanghai Pudong Hospital, Fudan University Pudong Medical Center Shanghai China; ^6^ Department of Vascular Surgery Huashan Hospital, Fudan University Shanghai China; ^7^ Department of Vascular Surgery Shanghai Pudong Hospital, Fudan University Pudong Medical Center Shanghai China; ^8^ Department of Neurology Zhongshan Hospital, Fudan University Shanghai China; ^9^ Institute of Healthy Yangtze River Delta Shanghai Jiao Tong University Shanghai China

**Keywords:** chronic cerebral hypoperfusion (CCH), interferon signaling, microglial activation, multiomics analysis, PU.1

## Abstract

Chronic cerebral hypoperfusion (CCH) is a significant factor that accelerates cognitive deterioration, yet the mechanisms of hippocampal microglial activation in this context remain unclear. Using an integrative multiomics approach, we investigated the transcriptional and epigenomic landscape of microglial activation in a mouse model of CCH induced by bilateral common carotid artery stenosis. Behavioral assessments revealed cognitive impairments, while neuropathological analysis confirmed hippocampal damage. Proteomic and transcriptomic profiling uncovered significant upregulation of stress and inflammatory pathways, particularly the interferon (IFN) signaling cascade. Epigenomic analysis identified regions of open chromatin, suggesting active transcriptional regulation driven by the transcription factor (TF) PU.1. ChIP‐nexus analysis further confirmed that PU.1 directly modulates the expression of IFN‐stimulated genes (ISGs), which are pivotal in regulating microglial activation. Our findings demonstrate that PU.1 serves as a key regulator of the IFN‐driven microglial response during CCH, mediated by enhanced chromatin accessibility and transcriptional activation of ISGs. This study highlights the critical role of PU.1 in microglial‐mediated neuroinflammation and offers potential therapeutic targets for mitigating hippocampal damage associated with chronic cerebral ischemia.

## Graphical abstract

In this study, we utilized a multiomics approach to investigate the response of hippocampal microglia to BCAS‐induced hypoperfusion. Proteomic, transcriptomic, and epigenomic analyses revealed that microglia are key responders to interferon (IFN) signaling. PU.1 underwent epigenetic modifications, correlating with altered chromatin accessibility and transcriptional regulation of genes driving microglial activation and inflammation during chronic cerebral ischemia.

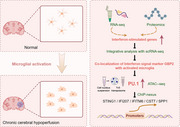

## 1 Introduction

Chronic cerebral hypoperfusion (CCH), a common feature of neurodegenerative and cerebrovascular diseases, is characterized by sustained reductions in cerebral blood flow (CBF), leading to energy deficits, oxidative stress, and neuronal damage [1, 2, 3]. These pathological changes primarily affect the hippocampus, contributing to cognitive impairments observed in conditions such as Alzheimer's disease (AD) and vascular cognitive impairment (VCI) [4]. Neuroimaging studies have revealed reduced glucose metabolism in the hippocampus and other critical brain regions in CCH, underscoring its role in driving disease progression [5]. However, the molecular mechanisms underlying hippocampal damage in CCH remain incompletely understood.

CCH pathophysiology involves complex processes, including blood–brain barrier disruption, neuroinflammation, oxidative stress, endothelial dysfunction, and apoptosis. Among these, chronic neuroinflammatory responses driven by activated microglia are pivotal [6, 7]. Prolonged ischemia induces a pro‐oxidative microglial phenotype, resulting in the secretion of inflammatory mediators such as TNF‐α, IL‐1β, COX‐2, and iNOS [8]. This sustained inflammatory state exacerbates neuronal damage and disrupts brain homeostasis. Additionally, microglia regulate central nervous system cell fate through intercellular signaling, highlighting the need to dissect the mechanisms underlying microglial activation and morphological changes in CCH [9].

Emerging evidence points to the critical involvement of the cyclic GMP–AMP synthase (cGAS) pathway in neuroinflammation during ischemic injury. cGAS activation triggers interferon (IFN)‐regulated gene expression, amplifying the release of pro‐inflammatory cytokines and exacerbating ischemic brain damage [10]. Our recent RNA sequencing (RNA‐seq) analysis in a bilateral common carotid artery stenosis (BCAS) model revealed significant transcriptomic changes in the hippocampus, with a notable enrichment of type I IFN (IFN‐I) signaling pathways [11]. While these findings underscore the role of IFN‐I in CCH, upstream regulatory mechanisms remain inadequately explored.

Recent advancements in multiomics approaches, including transcriptomics, proteomics, and epigenomics, have provided transformative insights into the molecular underpinnings of brain disorders [12, 13]. For example, ATAC‐seq (Assay for Transposase‐Accessible Chromatin using sequencing) enables the identification of regulatory chromatin regions that influence gene expression, offering a deeper understanding of transcriptional control mechanisms [14]. Multiomics integration has proven effective in uncovering regulatory networks and disease‐specific molecular markers, facilitating precision medicine strategies [15, 16]. Notably, epigenomic studies of aging microglia have revealed profound changes in transcriptional regulation and chromatin accessibility, emphasizing the utility of these approaches in dissecting complex pathological processes [17, 18].

In the context of CCH, PU.1, a master regulator of microglial function, has been implicated in controlling chromatin accessibility and the transcription of immune‐related genes [19]. While its role in microglial biology is well‐documented, its contribution to postischemic microglial responses remains underexplored.

In this study, we applied a multiomics framework to investigate hippocampal alterations in a BCAS‐induced CCH model. Behavioral deficits and neuropathological changes were accompanied by transcriptomic and proteomic evidence of IFN pathway activation. Epigenomic profiling identified PU.1‐regulated chromatin regions associated with microglial activation and IFN‐stimulated gene (ISG) expression. These findings highlight PU.1 as a key mediator of microglial responses to ischemic injury and underscore the potential of multiomics approaches to identify novel therapeutic targets for CCH‐induced cognitive impairments.

## 2 Results

### 2.1 CBF Reduction, Behavioral Impairments, and Neuropathological Changes in BCAS‐Induced Chronic Cerebral Ischemia

**FIGURE 1:**
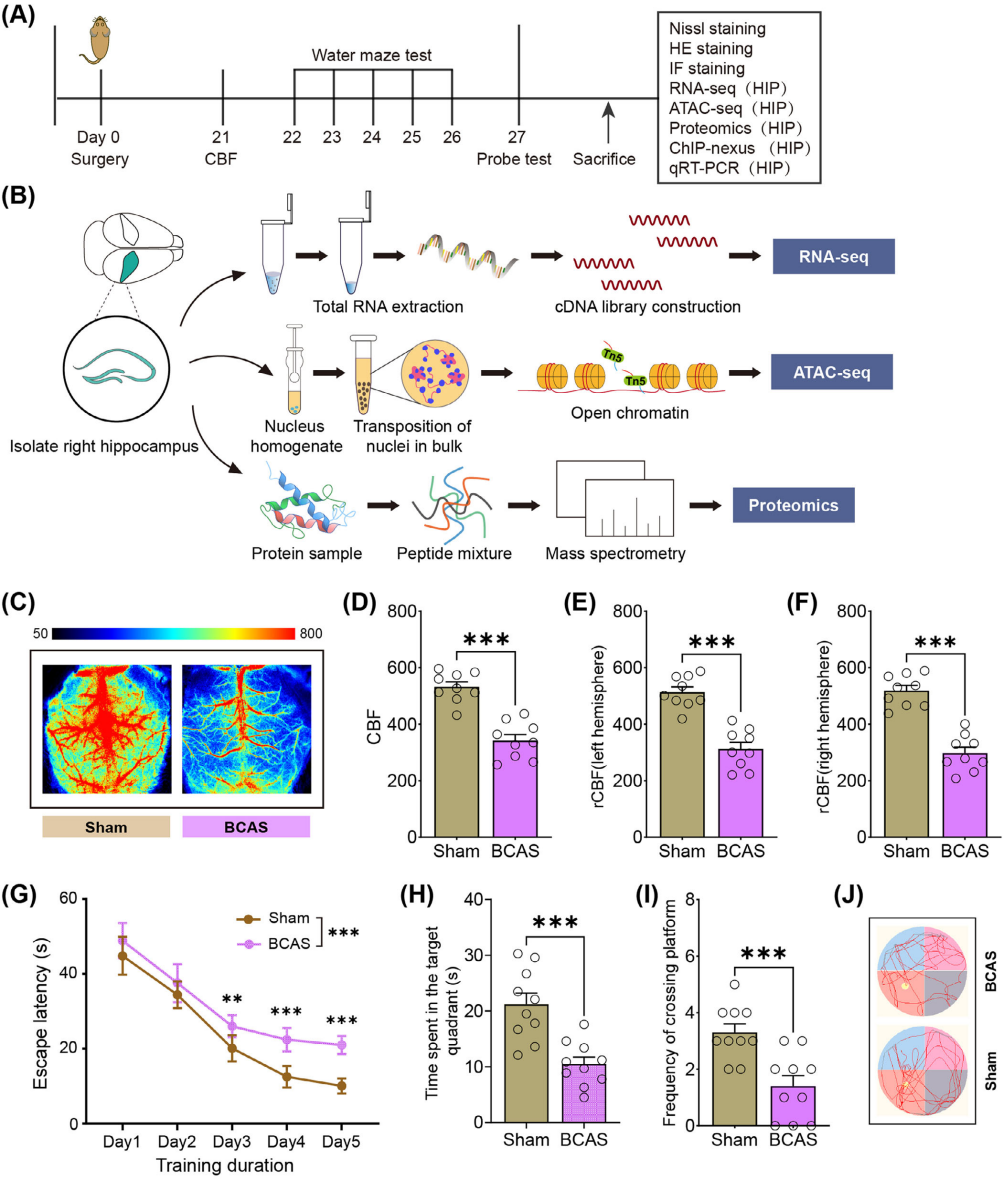
Alterations in CBF and behavioral outcomes after BCAS‐operation. (A) Experimental flowchart. IF, immunofluorescence; ChIP, chromatin immunoprecipitation; HIP, hippocampus. (B) Workflow for hippocampus‐specific RNA‐seq, ATAC‐seq, and proteomics experiments. (C) Representative laser speckle images of CBF measured 3 weeks post‐BCAS or sham surgery. Bar charts showing CBF for the whole brain (D), left hemisphere (E), and right hemisphere (F). (G) Escape latency comparison during the MWM learning phase (two‐way ANOVA). (H and I) Time spent in the target quadrant and number of platform crossings in the probe test. (J) Tracking plots showing movement patterns in the MWM probe test for sham and BCAS mice. Data are expressed as mean ± SEM. Each group consisted of 9–10 mice. Statistical significance, **p < 0.01, ***p < 0.001.

The experimental workflow is depicted in Figure 1A,B. Three weeks postinduction of hypoperfusion via BCAS, CBF was significantly reduced (Figure 1C–F). Specifically, the use of 0.16/0.18 mm microcoils led to a 42.6% decrease in mean CBF compared with the ipsilateral hemisphere of sham‐operated controls (Figure 1F). These findings are consistent with previous reports of BCAS‐induced hypoperfusion [20]. To assess the impact of chronic ischemia on cognitive function, spatial learning and memory were evaluated using the Morris Water Maze (MWM). BCAS mice exhibited pronounced deficits in spatial learning during the training phase compared with sham‐operated mice (Figure 1G). During the probe trial, BCAS mice spent significantly less time in the target quadrant and showed reduced platform crossings (Figure 1H–J), indicating reference memory impairment. Together, these data demonstrate that BCAS‐induced cerebral hypoperfusion leads to significant cognitive impairments following 3 weeks of surgery.

To investigate neuropathological alterations, histological analyses of hippocampal sections were performed using Nissl and hematoxylin and eosin (HE) staining. Severe hypoperfusion caused by 0.16 mm microcoil stenosis resulted in hippocampal atrophy and marked neuronal loss, particularly in the CA1 region (Figure S1A–C). Quantification of Nissl‐stained sections confirmed a significant reduction in neuronal density in the CA1 area on the 0.16 mm microcoil‐treated side of BCAS mice (Figure S1B). Microglial activation was examined via Iba1 (microglia) immunostaining. Substantial microglial activation was observed in infarcted hippocampal regions, particularly in the CA1 and CA3 areas of the 0.16 mm microcoil‐treated hemisphere (Figure S1D,E). Quantitative analysis revealed significantly higher microglial activation on the 0.16 mm side compared with the 0.18 mm side (Figure S1E). Costaining of Iba1 and NeuN (neurons) confirmed a strong association between microglial activation and neuronal degeneration in areas affected by severe hypoperfusion (Figure S1F–H).

These findings highlight the critical role of microglial activation in the inflammatory response to chronic cerebral ischemia and its association with hippocampal neuronal degeneration. The results provide insights into the cellular dynamics of ischemia‐induced neuroinflammation and underscore the importance of targeting microglial activation in therapeutic strategies for ischemic brain injury.

### 2.2 Hippocampal Proteomic Alterations Induced by BCAS‐Driven Hypoperfusion

To elucidate molecular changes associated with BCAS‐induced chronic cerebral ischemia, we performed proteomic profiling specific to the hippocampus. Quality control metrics indicated robust and reliable data (Figure S2). A total of 6,885 proteins were identified, of which 6817 were shared between the sham and BCAS groups, representing 99% of the total proteome and underscoring the consistency of our dataset (Figure S3A). Correlation heatmaps revealed clear segregation between the sham and BCAS groups, with high intragroup consistency, supporting strong biological reproducibility (Figure S3B). Differentially expressed proteins (DEPs) were identified using criteria of |log2FC| > 1 and *p* < 0.05. A total of 317 DEPs were detected, with 281 (88.6%) upregulated and 36 (11.4%) downregulated in the BCAS group compared with controls (Figure S3C and Table S2). Cluster heatmaps indicated that the majority of upregulated proteins were enriched in the BCAS group (Figure S3D). Gene Ontology (GO) enrichment analysis of DEPs revealed significant activation of immune‐related biological processes, including “Antigen processing and presentation of endogenous peptide antigen,” “Macrophage activation,” “Cellular response to interferon‐beta,” and “Astrocyte activation” (Figure S3E,F). Conversely, downregulated processes were associated with “RNA catabolic process,” “Synaptic signaling,” and “Synaptic transmission,” implicating impaired neuronal communication and metabolic regulation in BCAS‐induced hypoperfusion (Figure S3G).

These findings provide critical insights into the molecular mechanisms underlying hippocampal dysfunction during chronic cerebral ischemia. The enrichment of immune‐related pathways and suppression of synaptic processes highlight the dual roles of inflammation and neuronal dysregulation in the progression of ischemic brain injury, offering potential targets for therapeutic intervention.

### 2.3 Integrative Transcriptomic and Proteomic Analyses Highlight IFN‐I Pathway Activation

**FIGURE 2:**
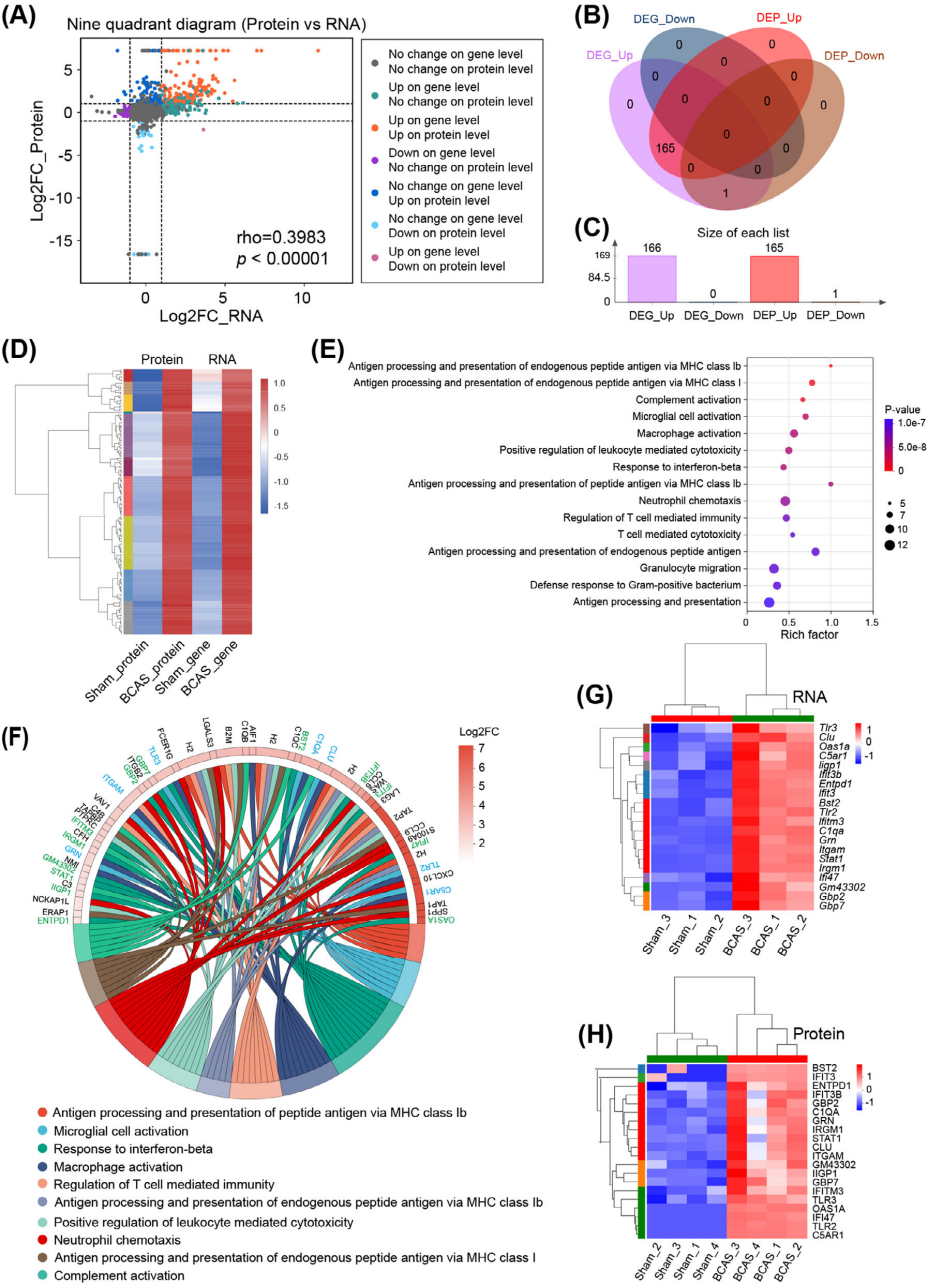
Combined transcriptome and proteome analyses of hippocampus post BCAS hypoperfusion. (A) Nine‐quadrant diagram for the transcriptome‐proteome correlations. (B) The Venn diagram of two groups for up‐ and downregulated differential expressed genes (DEGs) and DEPs. BCAS versus Sham. (C) The number of DEGs and DEPs statistics between sham and BCAS groups. Note that 165 common differential genes were identified. (D) Cluster heat maps of the 165 co‐upregulated DEGs and DEPs. (E) GO functional enrichment analysis of the 165 differential genes. (F) Chord diagram of top 10 enriched GO terms and related differential genes. Notice the “microglial cell activation pathway” genes marked in blue font and the “response to interferon‐beta” pathway genes marked in green font. (G and H) Clustering heat map of marker genes in “Microglial cell activation pathway” and “Response to interferon‐beta pathway” from transcriptome and proteome respectively.

To explore the molecular mechanisms underlying BCAS‐induced hippocampal dysfunction, we integrated transcriptomic and proteomic data. Correlation analysis revealed a strong association between mRNA and protein expression levels for differentially expressed genes (DEGs) in the hippocampus (Figure 2A). Venn diagram analysis identified 165 genes upregulated at both the transcript and protein levels, demonstrating a high degree of overlap between these datasets (Figure 2B,C and Table S3). Cluster heatmaps further confirmed the tight correlation between RNA and protein expression for these coupregulated genes (Figure 2D). GO enrichment analysis of the 165 shared DEGs revealed significant enrichment in pathways related to immune response, including “Antigen processing and presentation,” “Complement activation,” “Microglial/macrophage activation,” and “Interferon signaling” (Figure 2E). A chord plot visualized the top 10 enriched GO terms, highlighting key genes involved in the “Microglial/macrophage activation pathway” (e.g., *Itgam, C5ar1, Tlr2, Tlr3, C1qa, Grn*) and “IFN‐related pathways” (e.g., *Irgm1, Oas1a, Ifit3, Gbp2, Stat1*) (Figure 2F). Cluster heatmaps indicated these pathways were strongly upregulated at both transcript and protein levels in BCAS mice (Figure 2G,H).

To validate these findings, RT‐qPCR was performed on hippocampal tissues, confirming significant upregulation of genes associated with microglial activation (*Trem2, Tlr2, Aif1, C1qa, C5ar1*) and IFN signaling (*Gbp2, Irgm1, Ifitm3, Bst2*) in BCAS mice compared with sham controls (Figure S4A). Among these, GBP2, a key protein in the IFN‐β signaling pathway [21], was examined further using immunofluorescence. GBP2 expression was markedly increased in the hippocampus of BCAS mice, with confocal microscopy confirming its colocalization with activated microglia (Figures S5 and S4B,C).

These results suggest that BCAS‐induced chronic cerebral ischemia leads to distinct activation of immune‐related pathways, particularly involving microglial responses and IFN‐I signaling. The upregulation of IFN‐related genes and proteins in microglia underscores their central role in the pathophysiological response to chronic hypoperfusion. These findings provide critical insights into the molecular basis of neuroinflammation in chronic cerebral ischemia and identify the IFN pathway as a potential therapeutic target.

### 2.4 Identification of Hippocampus‐Specific Open Chromatin Regions by Epigenomics Analysis and Validation of the PU.1 Regulator in Ischemic Microglial Activation

**FIGURE 3:**
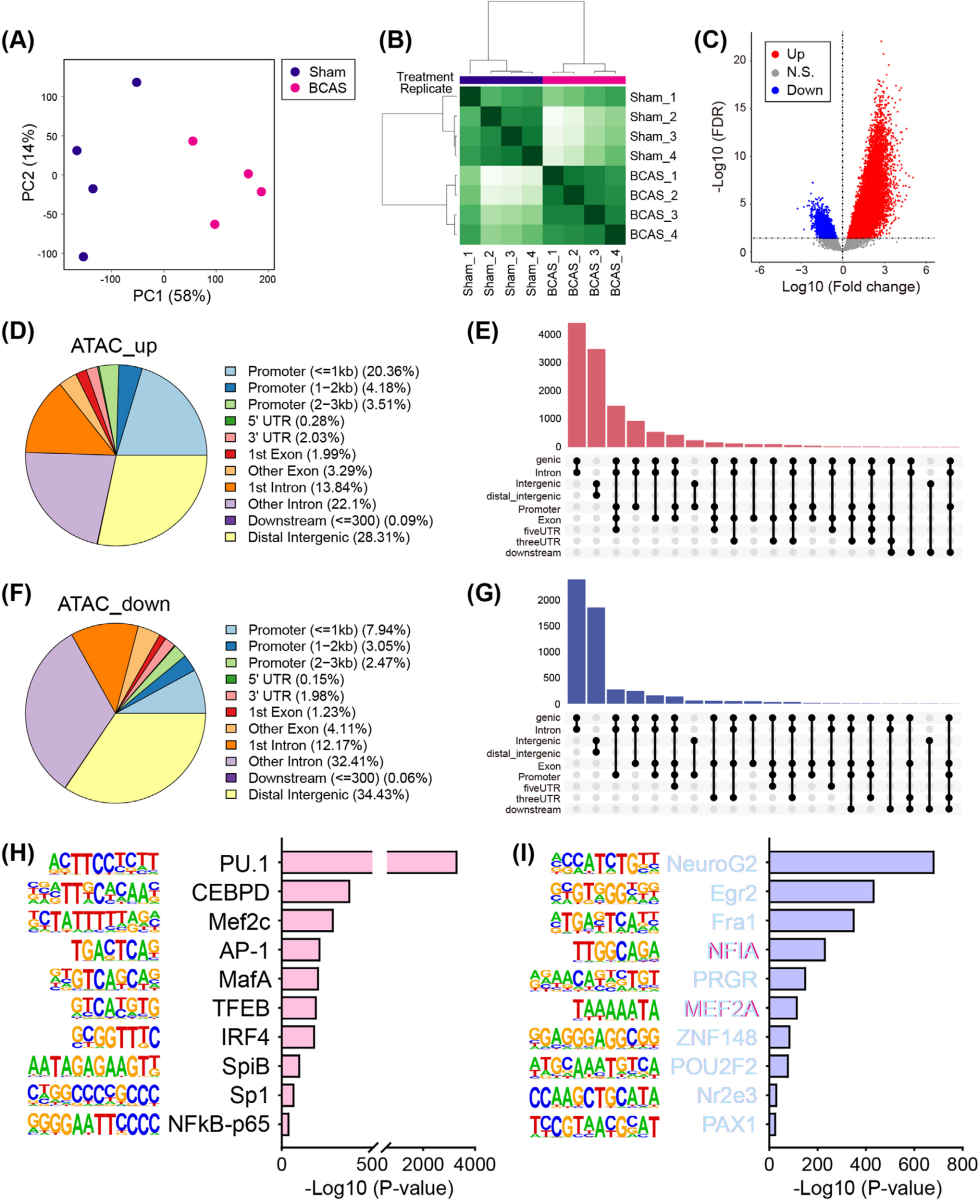
Alterations in chromatin accessibility in the hippocampus following BCAS‐induced hypoperfusion. (A) Principal component analysis (PCA) of ATAC‐seq data from hippocampal samples in sham and BCAS groups (*n* = 4 per group). (B) Heatmap showing correlation analysis between the two groups. (C) Volcano plot illustrating differentially accessible regions (DARs) in ATAC‐seq signals post‐BCAS hypoperfusion. (D) Annotations of genomic regions for upregulated ATAC‐seq loci. (E) UpSet plot showing intersections of accessible chromatin regions for upregulated loci across various genomic features. (F) Annotations of genomic regions for downregulated ATAC‐seq loci. (G) UpSet plot of intersections for downregulated loci across genomic features. (H and I) Homer de novo motif analysis predicting transcription factor motifs for up‐ and downregulated loci.

Given the observed gene expression changes following chronic cerebral ischemia, we conducted ATAC‐seq to map accessible chromatin regions, represented by ATAC‐seq peaks, in hippocampal tissue and to identify associated TFs. The related quality control results were summarized in Figures S6 and S7. Principal component analysis (PCA) revealed a distinct separation in ATAC‐seq signals between these two groups (Figure 3A). The heatmap further confirmed this separation, showing clear group clustering (Figure 3B). Subsequent analysis identified significant differentially accessible regions (DARs) in ATAC‐seq signals (Figure 3C and Table S4). The majority of these loci, whether upregulated or downregulated, were mapped to noncoding regions: “ATAC_up” included 28.3% distal intergenic, 28.1% promoter, and 35.9% intron, while “ATAC_down” included 34.4% distal intergenic, 13.5% promoter, and 44.6% intron (Figure 3D–G). DARs were more abundant in genic‐intron regions than intergenic regions. Transcription factor (TF) motif enrichment analysis using HOMER showed strong enrichment of ETS family motifs in hippocampus‐specific peaks (Figure 3H,I).

**FIGURE 4:**
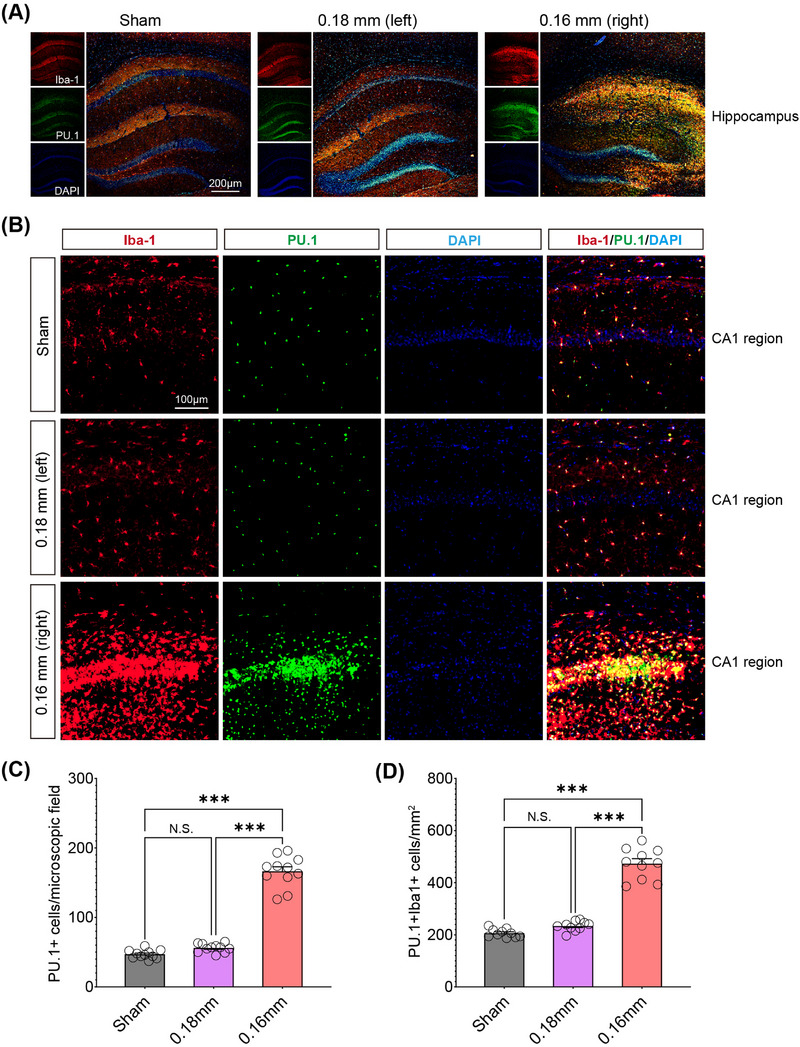
Elevated number and morphological activation of Iba1+PU.1+ microglia are evident in the hippocampal area post BCAS‐induced hypoperfusion. (A) Immunofluorescence analysis showing PU.1/Iba1 costaining of the hippocampus in both sham‐ and BCAS‐operated groups. (B) Representative images of PU.1/Iba1 costaining in the CA1 region of the mouse hippocampus. (C and D) Quantification of the number of PU.1‐positive cells per microscopic field and PU.1‐Iba1 double‐positive cells respectively. Data are expressed as mean ± SEM. One‐way ANOVA test, NS = not significant, ***p ˂ 0.001.

Among the “ATAC_up” loci, the PU.1 motif showed the highest level of enrichment, followed by CEBPD and MEF2C. Given the central role of microglia in neuroinflammation, the pronounced increase in PU.1 motif enrichment could indicate an enhanced immune response, which may exacerbate or modulate the damage caused by chronic hypoperfusion. Immunofluorescence showed that cerebral hypoperfusion increased PU.1 expression in the hippocampus of the BCAS group (Figure 4A–C). Colocalization with activated microglia was noted, and quantitative analysis revealed significantly more PU.1+Iba1+ cells on the 0.16 mm microcoil‐treated side of the BCAS group (Figure 4D). These results suggest that microglial PU.1 may play a key role in regulating immune responses to chronic cerebral ischemia.

### 2.5 Multiomics Profiling Identifies Microglia as the Key Mediators of IFN Responses in BCAS‐Induced Hypoperfusion

**FIGURE 5:**
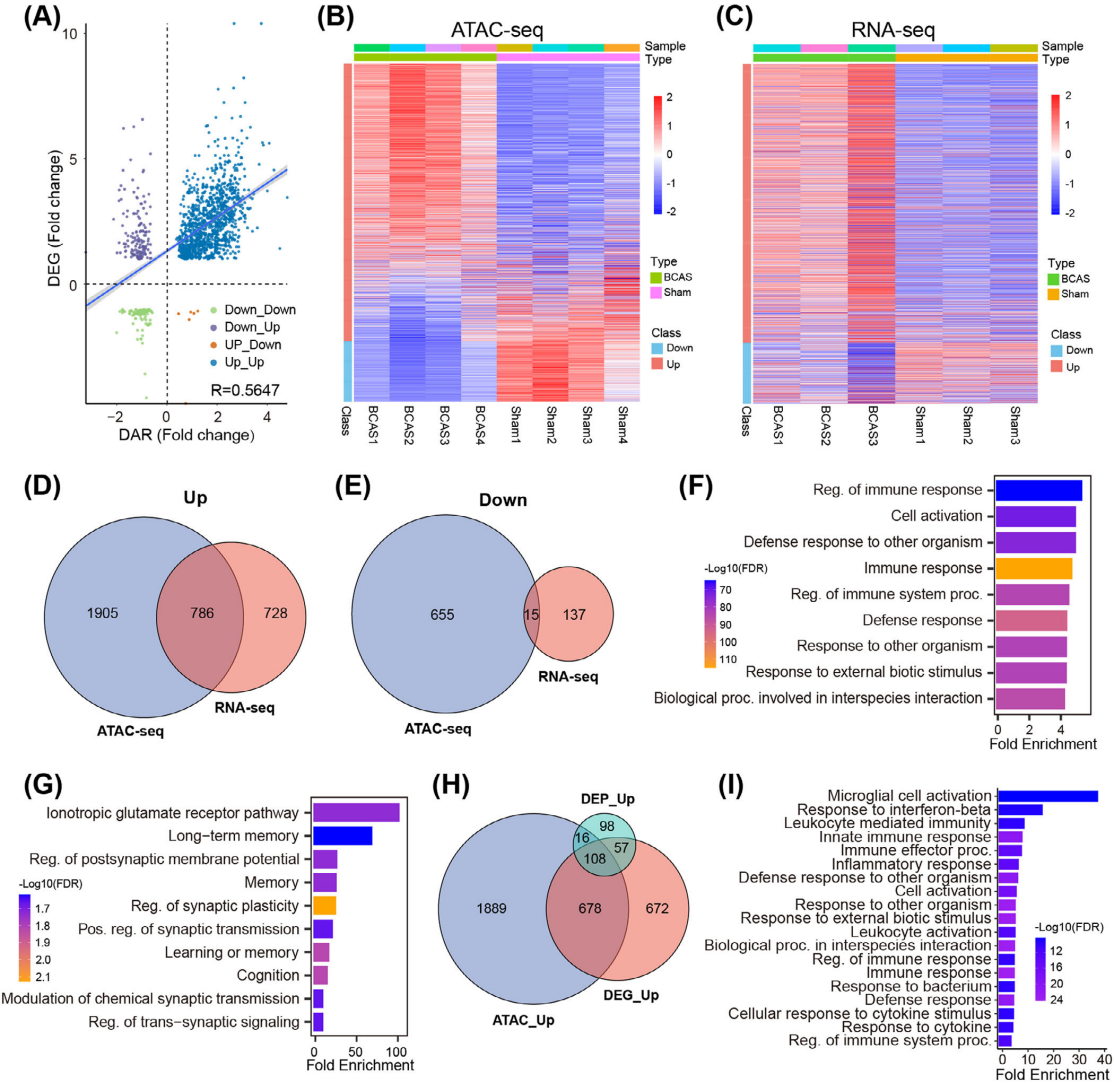
Multiomics profiling of hippocampus‐specific RNA‐seq, ATAC‐seq, and proteomics data following BCAS hypoperfusion. (A) Correlation analysis between gene expression changes and chromatin accessibility. (B) Expression changes of DEGs mapped onto ATAC‐seq signals. (C) Heatmap of DEGs identified by RNA‐seq. (D and E) Venn diagrams illustrating common upregulated and downregulated genes identified in both RNA‐seq and ATAC‐seq datasets. (F) Top 9 enriched GO terms for upregulated genes shared between DAR‐related genes and DEGs. (G) Top 10 enriched GO terms for downregulated genes shared between DAR‐related genes and DEGs. (H) Venn diagram showing overlap among upregulated DAR‐related genes, DEGs, and DEPs, identifying 108 shared genes. (I) GO functional enrichment analysis of the 108 shared upregulated genes.

Integrative analysis of transcriptomic and epigenomic datasets revealed a strong correlation between gene expression and promoter accessibility, with most DEGs exhibiting increased accessibility in BCAS‐induced hypoperfusion (Figure 5A–C). Upregulated genes showed significantly higher promoter accessibility compared with downregulated genes, highlighting the influence of chromatin dynamics on transcriptional regulation. A combined analysis of differential ATAC‐seq peaks and RNA‐seq data identified 786 codifferentially expressed genes, predominantly upregulated (Figure 5D,E and Table S5 and S6). Functional enrichment analysis of these genes revealed significant involvement in immune processes, including “Regulation of immune response,” “Cell activation,” and “Defense response” (Figure 5F). In contrast, downregulated genes were enriched in neuronal signaling pathways such as “Long‐term memory,” “Regulation of postsynaptic membrane potential,” and “Regulation of synaptic plasticity” (Figure 5G). Cross‐omics integration of transcriptome, proteome, and epigenome datasets further identified 108 upregulated genes shared across all three omics layers (Figure 5H). These genes were enriched in pathways such as “Microglial activation,” “Interferon‐stimulated signaling,” and “Innate immune response” (Figure 5I), underscoring microglia's central role in the inflammatory response to chronic ischemia.

**FIGURE 6:**
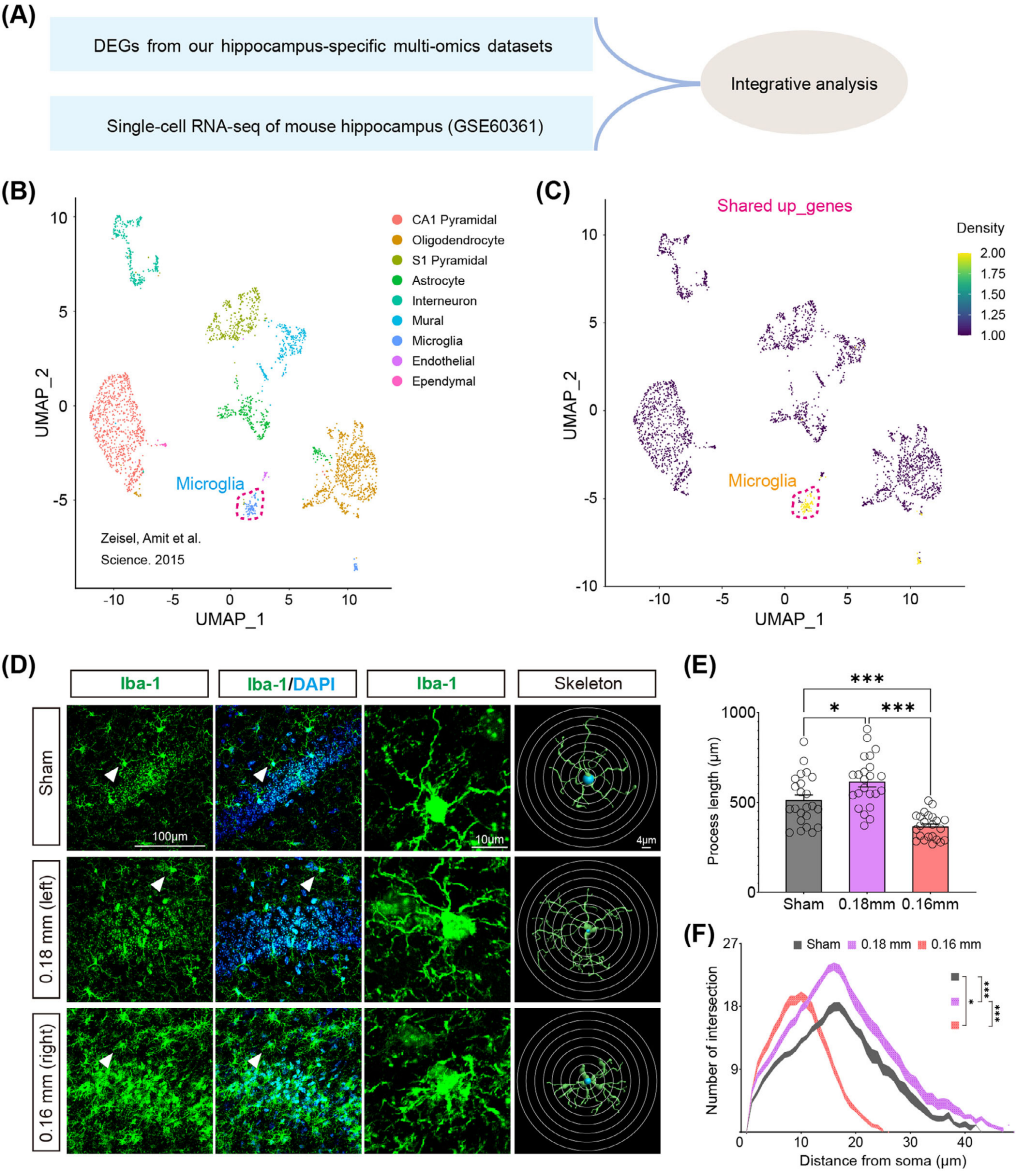
Integration of single‐cell RNA sequencing (scRNA‐seq) data reveals hippocampus‐specific upregulated genes predominantly enriched in microglia. (A) Schematic representation of the analytical approach. (B) UMAP plot visualizing the clustering of single cells by cell type, based on previously published scRNA‐seq data (Zeisel et al., 2015). (C) Density scatterplots generated using the *UCell* R package show cell‐type enrichment for “Up_genes.” Upregulated genes are notably enriched in the “Microglia” cluster. (D) Representative immunofluorescence images of anti‐Iba1 staining in the hippocampal CA1 region from sham and BCAS groups. (E) Bar plot quantifying microglial process lengths, analyzed with one‐way ANOVA (*N* = 23 processes/6 animals/group). (F) Sholl analysis evaluating microglial branch complexity was performed using two‐way ANOVA (*N* = 23 processes/6 animals per group). Data are shown as mean ± SEM, with significance levels of *p < 0.05, ***p < 0.001.

Single‐cell RNA sequencing (scRNA‐seq) analysis using a publicly available dataset (GSE60361) stratified hippocampal cells into nine major types, including microglia, astrocytes, pyramidal neurons, and oligodendrocytes (Figure 6A,B) [22]. Enrichment analysis of CCH‐induced shared upregulated genes showed a dominant association with microglia (Figure 6C). Morphological analysis in the BCAS model revealed distinct microglial responses to ischemic severity: hyper‐ramified microglia with increased branching were observed under mild ischemia (0.18 mm microcoil), while amoeboid‐like microglia with shorter branches and fewer intersections dominated in more severe ischemia (0.16 mm microcoil) (Figure 6D–F). These findings suggest that microglial activation varies depending on the extent of hypoperfusion, with milder ischemia promoting surveillance functions and severe ischemia driving an inflammatory response.

Proteomic analysis and immunofluorescence experiments corroborated these findings. CD68/Iba1 double staining revealed an increased proportion of phagocytic microglia (CD68+Iba1+) in the severely ischemic hemisphere (0.16 mm) compared with the mildly ischemic side (Figure S8). These activated microglia exhibited distinct phenotypes, including upregulation of IFN‐related signaling pathways.

Collectively, these results establish microglia as the primary cellular mediators of IFN responses in chronic ischemic injury. Their transcriptional, proteomic, and morphological adaptations underscore their central role in driving inflammation and tissue remodeling, offering new insights into potential therapeutic targets for chronic cerebral ischemia.

### 2.6 PU.1 Drives Transcriptional Upregulation of ISGs in Microglial Activation During BCAS‐Induced Hypoperfusion

**FIGURE 7:**
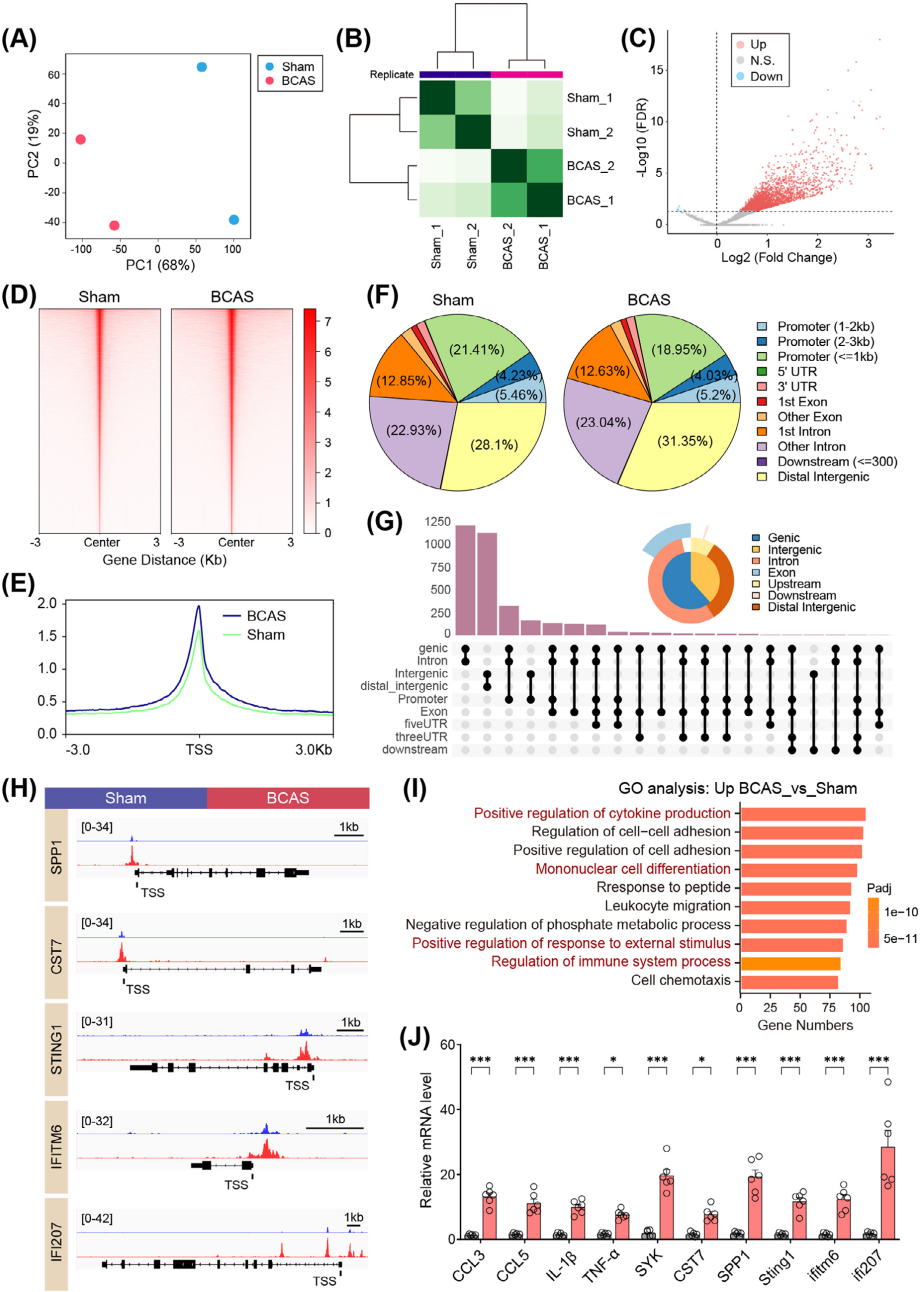
ChIP‐nexus analysis reveals that PU.1 promotes the transcriptional upregulation of interferon‐stimulated genes. (A) PCA analysis of hippocampus‐specific ChIP‐nexus data, with each group comprising two samples. (B) Heatmap displays the correlation analysis between these two groups. (C) The volcano plot identifies PU.1 binding peaks of the hippocampal ChIP‐nexus analysis after BCAS‐induced hypoperfusion. Upregulated and downregulated peaks are marked in red and blue, respectively, with non‐significant regions shown in gray. (D) The binding density of PU.1 was visualized with deepTools. (E) Metagene analyses of PU.1 coverages at transcription start sites (TSS). Regions selected from TSS (±3 kb). (F) Genome wide distribution of PU.1 binding peaks in sham‐ and BCAS‐treated groups, respectively. (G) Upset plot illustrating the overlap of accessible regions for upregulated PU.1 binding peaks across genomic features. BCAS versus Sham. (H) Genome browser tracks of ChIP‐nexus signal at the representative target gene loci. The black rectangles indicate the up‐peak regions of PU.1 on target‐gene promoters. (I) GO analysis of the elevated PU.1 binding peaks at candidate target genes. Notice that upregulated genes predominantly engage in biological processes, as depicted in the histogram. (J) Quantification of mRNA expression for identified genes (*Ccl3*, *Ccl5*, *Il‐1β*, *Tnf‐α*, *Syk, Cst7*, *Spp1*, *Sting1*, *Ifitm6*, *Ifi207*) in sham and BCAS groups, as measured by RT‐qPCR. Data are presented as mean ± SEM. Unpaired two‐tailed t‐test was used for statistical analysis, *p < 0.05, ***p < 0.001. *N* = 6 mice per group.

PU.1, a key TF regulating microglial activation, exerts its influence through epigenetic modifications that enhance chromatin accessibility and promote gene transcription [23]. Multiomics analysis revealed that BCAS‐induced hypoperfusion leads to substantial changes in chromatin accessibility, particularly in microglial cells. Given the critical role of IFN‐I signaling in microglial activation during ischemic brain injury, we investigated whether PU.1 directly regulates the transcription of ISGs under conditions of CCH. ChIP‐nexus analysis was conducted to identify the downstream target genes of PU.1. PCA showed a clear separation in ChIP‐nexus signals between the sham and BCAS groups (Figure 7A). The heatmap further highlighted distinct clustering within each group (Figure 7B). Subsequent analysis identified significantly altered ChIP‐nexus peaks (Figure 7C). Most of these peaks were located within 3 kb of the TSS (promoter region) (Figure 7D–G). Integrative analysis highlighted several upregulated genes associated with neuroinflammation and IFN‐I signaling in BCAS mice, including *Cst7* (2.0‐fold), *Spp1* (2.3‐fold), *Tlr2* (1.9‐fold), *Ccl7* (2.3‐fold), *Ccl12* (3.2‐fold), *Ifi207* (1.7‐fold), *Sting1* (1.5‐fold), and *Ifitm6* (2.6‐fold) (Figures 7H and S9). These genes were significantly enriched in immune response‐related pathways, such as cytokine production, cell–cell adhesion, mononuclear cell differentiation, and regulation of immune system processes, as revealed by GO analysis (Figure 7I).

**FIGURE 8:**
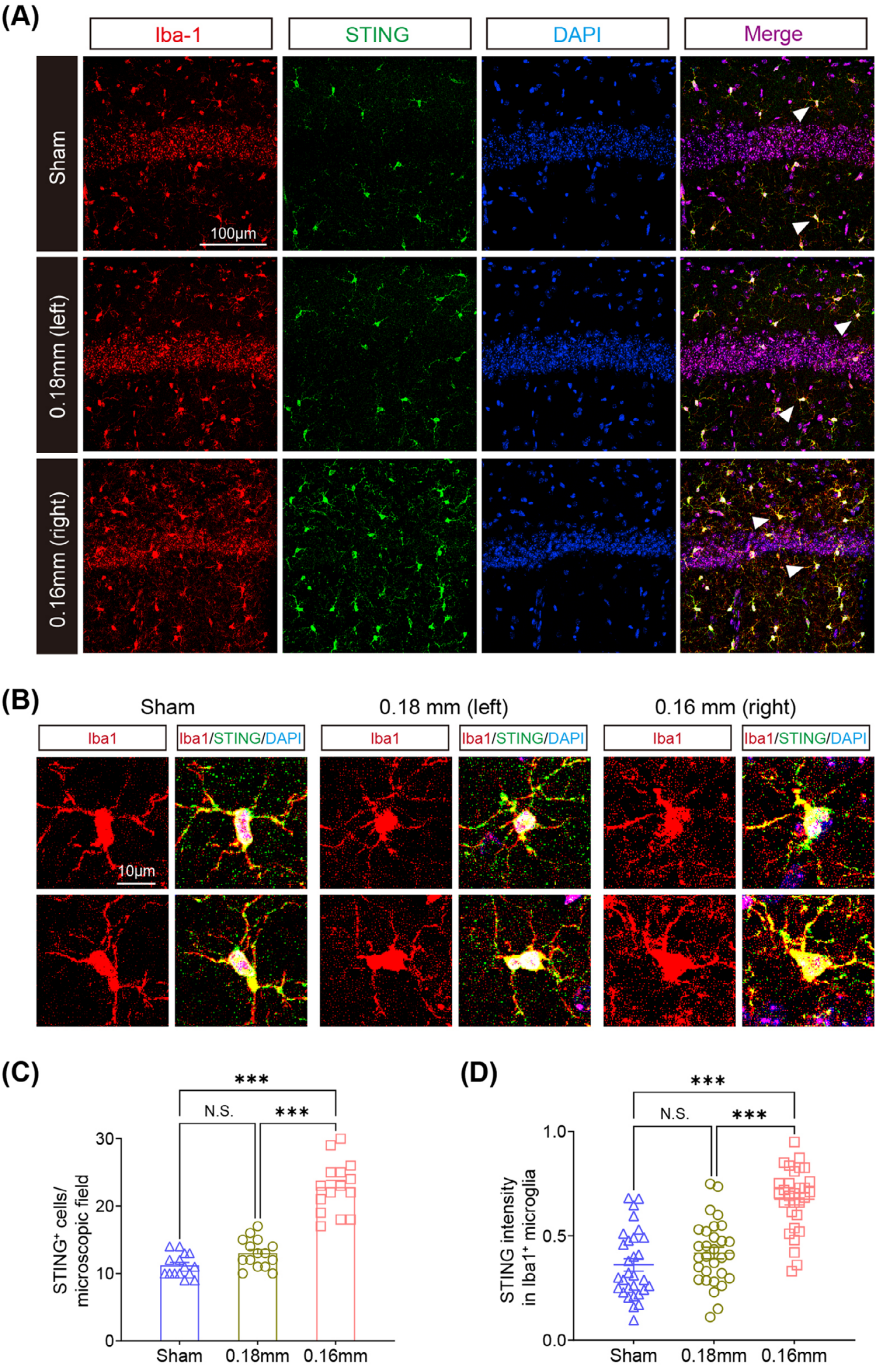
Stimulator of interferon genes (STING) was downstream targets of PU.1. (A and B) High‐resolution images of Iba1/STING immunofluorescence revealed increased microglia positive for STING (indicated by arrowheads) in BCAS mice. Notice that the importance of STING in microglial activation. (C and D) Quantification of the number of STING‐positive cells per microscopic field (*n* = 15) and STING intensity (*n* = the number of Iba1+ cells, *n* > 30 cells per group) respectively. Data are expressed as mean ± SEM. One‐way ANOVA, NS = not significant, ***p ˂ 0.001.

To validate these findings, RT‐qPCR confirmed elevated expression of these candidate genes in hippocampal tissue from BCAS mice, consistent with transcriptomic and ChIP‐nexus analyses (Figure 7J). Immunofluorescence further demonstrated that STING, a key mediator of IFN signaling, was highly upregulated and colocalized with activated microglia (Iba1+) in the hippocampus of BCAS mice, while its expression was minimal in sham controls (Figures 8A–D and S10).

These findings highlight PU.1 as a critical regulator of ISG transcription during microglial activation in chronic ischemic injury. By modulating chromatin accessibility and promoting the transcription of key immune response genes, PU.1 orchestrates the pro‐inflammatory microglial phenotype associated with cerebral hypoperfusion. The interplay between PU.1 and ISGs, including STING and other IFN‐related pathways, underscores the central role of PU.1 in driving the epigenetic and transcriptional programs underlying neuroinflammation.

Overall, these results establish PU.1 as a master regulator of microglial responses to chronic ischemia, offering a mechanistic framework for understanding its role in modulating the immune landscape of the injured brain. Targeting PU.1 or its downstream pathways may provide novel therapeutic opportunities to mitigate inflammation and neurodegeneration in chronic cerebral ischemia.

## 3 Discussion

In this study, hippocampus‐specific proteomic and transcriptomic analyses revealed a strong enrichment of transcripts and proteins within the IFN signaling pathway, essential for the immune response under BCAS‐induced hypoperfusion. Epigenomic profiling further identified specific regions of open chromatin in the hippocampus, suggesting active transcriptional regulation. Our multiomics approach demonstrated that microglia are the primary responders to IFN signaling during BCAS hypoperfusion. Additionally, PU.1 underwent epigenetic modifications in response to chronic cerebral ischemia, correlating with altered chromatin accessibility. ChIP‐nexus analysis identified PU.1 downstream target genes, highlighting its role in driving microglial activation and the inflammatory response. This multilayered approach allowed us to integrate chromatin accessibility data with TF binding profiles and gene expression, providing a robust framework to explore the molecular mechanisms underlying microglial activation in ischemic conditions.

Rodent models have been instrumental in mimicking the pathological features of VCI, with CCH models in rats, gerbils, and mice proving effective for studying sustained ischemia. Among these, the BCAS mouse model is particularly relevant, closely replicating the pathological hallmarks of human VCI and serving as a key tool for investigating therapeutic strategies [24, 25, 26]. Previous studies demonstrated that bilateral 0.16 mm microcoils in the BCAS model significantly reduce CBF and cause severe gray matter lesions but are associated with a high mortality rate (∼75% within 14 days), limiting their utility. In contrast, using 0.18 mm microcoils results in moderate CBF reduction, lower mortality (∼15%), and predominantly white matter lesions, with minimal gray matter involvement [27]. To overcome these limitations, a modified BCAS model using asymmetric 0.16/0.18 mm microcoils was developed. This approach induces asymmetric ischemia between hemispheres, resembling clinical CCH conditions, while achieving a reduced mortality rate (∼20%) [28]. Importantly, this model produces diffuse infarctions and significant gray matter damage in the severely stenosed hemisphere, offering a more accurate representation of human pathology. In our study, the 0.16/0.18 mm BCAS model was validated through behavioral assessments and histological analyses. Chronic cerebral ischemia was successfully induced, as evidenced by microinfarctions and moderate infarctions 3 weeks postsurgery, consistent with previous findings [23, 29]. This time point was chosen based on prior research showing peak microglial activation and cytokine release during the mid to late stages of ischemic injury, followed by a gradual decline. The results confirm the utility of the asymmetric BCAS model for investigating the pathophysiology of CCH and microglial‐driven immune responses.

The specific functions and mechanisms of IFN‐I signaling in CCH are less understood. Previous studies have emphasized the importance of IFN‐I signaling in various brain disorder models, where it regulates the production of pro‐inflammatory molecules and mediates intercellular interactions [30, 31]. Notably, RNA‐seq analyses of microglia from traumatic brain injury (TBI) models showed marked enrichment of MHC class I antigen presentation signal and IFN‐I signal, suggesting a key role for IFN‐I signaling in microglial phenotypic changes following TBI [32]. Our findings align with these observations, emphasizing the necessity for cell‐type specific analyses to develop targeted therapies. In vitro and in vivo studies have underscored the significance of ISGs in microglial responses to hypoxia/hypoglycemia and ischemia/reperfusion [33]. Moreover, targeting the IFN signaling cascade or enhancing autophagic pathways in APOE4 microglia presents novel strategies to mitigate APOE4‐associated pathologies in AD [34]. In aged microglia stimulated with LPS, there was a notable overexpression of IFN‐related genes (*Ifitm1*, *Ifitm6*, *Ilr2*) and upregulation of genes within the IFN pathway (*Il‐1β*, *Ifitm2*, *Ccl5*, *Ifi205*) [17]. IFITM3, another IFN‐induced protein, has been identified as a key regulator of microglial reactivity to inflammation through IFN‐β signaling [35]. Additionally, the role of bridging integrator 1 in modulating IFN‐I responses and disease‐associated microglia phenotypes highlights the complex regulation of microglial activity.

Our study focused on guanylate‐binding proteins (GBPs), particularly GBP2, as key indicators of IFN signaling. GBP2 colocalized with Iba‐1 in the cerebral cortex of TBI models, implicating its role in microglial activation [36]. Its sustained differential expression across various postinjury time points suggests a persistent function in the response to brain injury [37]. Furthermore, GBP2 expression in subarachnoid hemorrhage‐induced early brain injury indicates its potential impact on neuronal function and survival [38]. Immunofluorescence in our study confirmed GBP2 colocalization with activated microglia, underscoring the critical role of IFN signaling in microglial activation during CCH. These findings align with prior studies, highlighting IFN signaling as a key mediator of reactive microglia in BCAS‐induced hypoperfusion and providing insights for IFN‐targeted therapeutic strategies.

PU.1, a key member of the ETS TF family, plays a fundamental role in defining microglial identity and mediating their immune functions [39, 40, 41]. Our findings suggest that the significant enrichment of PU.1 binding motifs in regions of increased chromatin accessibility following CCH points to its involvement in driving microglial activation under ischemic conditions. Prior research has established PU.1 as essential for microglial maturation and response, implying that its upregulation could underlie the gene expression shifts seen during ischemic injury [42, 43]. In addition, the transcriptional activation of PU.1 is facilitated through interactions with ICSBP and IRF‐1, suggesting a synergistic effect with IFN‐I signaling in regulating immune‐inflammatory responses [44, 45]. The heightened presence of PU.1 motifs likely reflects its regulatory influence on genes tied to inflammation, phagocytosis, and tissue repair in response to ischemia. This suggests an intensified immune activity that may contribute to the progression of ischemic damage or serve in tissue remodeling. The strong association of PU.1 with these pathways underscores its potential as a therapeutic target for modulating microglial responses in the context of ischemic brain injury.

The stimulator of IFN genes (STING) pathway has been identified as a crucial mediator in the process of neuroinflammation, particularly influencing microglial activation and polarization [46, 47]. STING, an endoplasmic reticulum‐associated protein, detects cytosolic DNA and initiates a robust immune response through the activation of IFN‐I and other inflammatory cytokines [48]. In the context of ischemic stroke, STING expression is upregulated in microglia, particularly in response to mitochondrial DNA released from damaged cells [49]. This activation promotes the polarization of microglia toward the M1 phenotype, enhancing the inflammatory response and potentially worsening neuronal damage. Inhibiting the STING pathway has shown promising results in reducing ischemic damage. Pharmacological inhibitors such as C‐176 and H151 effectively diminish brain infarction, edema, and neuronal injury by attenuating the activation of downstream signaling molecules like IRF3 and NF‐κB, which are pivotal for M1 microglial responses [50, 51]. Additionally, our ChIP‐nexus analysis has revealed that several downstream target genes of PU.1, including STING, play significant roles in regulating IFN signaling pathways. This discovery positions PU.1 as not only a key upstream regulator of microglial activation but also as an integral component linking PU.1 to the IFN‐I response. This connection drives the cascade of immune‐inflammatory events following cerebral hypoperfusion, underscoring the complexity and importance of these pathways in the context of ischemic brain injury. Therefore, by elucidating the mechanisms through which STING modulates microglial responses, we gain valuable insights into potential therapeutic targets for mitigating neuroinflammation and promoting neuroprotection in ischemic brain injury. Future research should continue to explore the therapeutic potential of STING inhibitors and their role in the broader landscape of CCH treatment and recovery.

The novelty of this study lies in its integrative multiomics approach, combining transcriptomics, proteomics, and epigenomics to comprehensively analyze the molecular mechanisms underlying chronic cerebral ischemia. While this study offers important insights into the roles of PU.1 and IFN signaling in chronic cerebral ischemia, several limitations should be acknowledged. First, future studies using conditional PU.1 knockout mice specifically in microglia could significantly enhance our understanding of PU.1's role in regulating neuroinflammation under CCH conditions. This approach would provide a more targeted investigation of PU.1's function in microglial activity and help to dissect its specific contributions to ischemia‐induced neuroinflammatory pathways. Additionally, the broader implications of PU.1‐mediated regulation in other neuroinflammatory conditions have not been comprehensively addressed, limiting the generalizability of our results. Last, verifying the interaction between PU.1 and its downstream targets, such as STING, would further strengthen our findings. Despite these limitations, our study enhances the understanding of ischemic brain injury and paves the way for exploring new therapeutic interventions.

## 4 Conclusions

PU.1 is pivotal in the microglial response to CCH, influencing gene expression via epigenetic modifications and interacting with IFN‐I signaling pathways. These findings deepen our understanding of the molecular mechanisms driving neuroinflammation and highlight potential therapeutic targets for brain disorders. Future research should focus on exploring the therapeutic potential of modulating PU.1 activity and IFN signaling to reduce the harmful effects of chronic cerebral ischemia.

## 5 Materials and Methods

### 5.1 Animals

Male adult C57BL/6 mice (22–25 g) from Vital River (Beijing) were randomly assigned to BCAS or sham groups. Of 143 mice, 17 did not survive the BCAS procedure. The remaining 126 mice were allocated for specific experiments: 18 for CBF assessments and frozen sections, 20 for MWM, 16 for paraffin section staining, eight for ATAC‐seq, 32 for proteomics, 20 for ChIP‐nexus, and 12 for RT‐qPCR. All experiments followed ARRIVE guidelines and were approved by the Department of Laboratory Animal Science, Fudan University (Approval Number: 2023‐MHFY‐23JZS).

### 5.2 BCAS Surgery

BCAS surgery was performed on 11‐week‐old mice under isoflurane anesthesia (3% induction, 1.5–2% maintenance) [52]. A 0.16 mm microcoil was applied to the right carotid artery, followed by a 0.18 mm microcoil on the left after 1 h. Sham‐operated mice underwent the same procedure without coil placement. Mice were kept on a 37°C heating pad during surgery.

### 5.3 Laser Speckle Contrast Imaging

Three weeks postsurgery, CBF was measured using the RFLSI III system. Mice were anesthetized with isoflurane, the skull exposed via a midline incision, and scanned. Temperature was maintained at 36.5–37.5°C throughout the procedure.

### 5.4 MWM Test

The MWM test included visible platform training, hidden platform trials over 5 days, and a probe trial on day 6 [53]. Performance metrics included platform crossings and time spent in the target quadrant. Mice failing visible platform trials were excluded.

### 5.5 HE Staining and Nissl Staining

Paraffin‐embedded brain sections (4 µm) were stained with HE or crystal violet (Nissl) using standard protocols [54]. Sections were imaged with a digital slide scanner, and staining was evaluated for structural changes.

### 5.6 Immunofluorescence Staining

Animals were anesthetized with intraperitoneal pentobarbital (150 mg/kg) and transcardially perfused with PBS, followed by 4% PFA. Brains were then extracted, postfixed in 4% PFA for 16 h, and immersed in 15% sucrose for 24 h, followed by 30% sucrose for 48 h. Coronal sections (30 µm) were cut using a freezing microtome (CM 1900; Leica, Germany). For immunofluorescence, sections were washed with PBS, permeabilized with 1% Triton X‐100, and blocked for 1 h. The sections were incubated overnight at 4°C with the following primary antibodies: rabbit anti‐IBA1 (Abcam), mouse anti‐NeuN (Sigma), rat anti‐CD68 (Abcam), rabbit anti‐GBP2 (Abclonal), goat anti‐IBA1 (Abcam), rabbit anti‐PU.1 (CST), rat anti‐IBA1 (Abcam), and rabbit anti‐STING (Abcam). The next day, sections were washed and incubated with secondary antibodies (Alexa Fluor 594 or 488) for 1 h at room temperature, followed by DAPI staining for 15 min. Sections were imaged using an Olympus slide scanner (VS120), and microglial activation areas were manually defined and quantified using OlyVIA software. Confocal microscopy was used to capture high‐resolution images (Nikon AX Ti2E), and images were processed using ImageJ. Sholl analysis was conducted to assess microglial morphology, with 3D reconstructions created using Imaris software for detailed analysis of microglial process length.

### 5.7 Proteomics

Hippocampal samples were pooled (four mice per replicate), digested with trypsin, and analyzed via Orbitrap Astral mass spectrometry. Data‐independent acquisition (DIA) was used to identify and quantify proteins, with downstream analysis focusing on significantly DEPs.

### 5.8 ATAC‐seq Library Preparation and Sequencing

ATAC‐seq was conducted on freshly isolated hippocampal tissue. Samples were homogenized in lysis buffer with a dounce homogenizer and washed in sucrose buffer. Nuclei were isolated by centrifugation. Cell pellets were treated with 5 µL Tn5 enzyme in 50 µL transposition mix (Vazyme, TD501). DNA was then purified, eluted in 27 µL EB buffer, and amplified via PCR. The resulting products were purified using the VAHTS® DNA Clean Beads Kit (Vazyme). Library quality was checked with an Agilent 2100 TapeStation, and concentrations were measured with a QuBit 4 fluorometer (Invitrogen). The libraries were sequenced with 150 bp paired‐end reads on an Illumina NovaSeq 6000 system.

### 5.9 Extraction of mRNA and Quantitative Real‐Time PCR

Total RNA was isolated from right hippocampal tissue using the TRIzol extraction method, and any genomic DNA contamination was removed with an RNase‐free DNase set (QIAGEN). RNA concentration and purity were assessed using a NanoDrop 2000 spectrophotometer (Thermo). cDNA was synthesized from total RNA using the HiScript III All‐in‐one RT SuperMix Perfect for qPCR (Vazyme; R333). qPCR was then performed on a QuantStudio 5 system (Thermo) with ChamQ Universal SYBR qPCR Master Mix (Vazyme; Q711). Primer sequences are provided in Table S1. *Gapdh* was used as the reference gene, and relative mRNA levels were calculated using the 2^−∆∆CT^ method.

### 5.10 Preparation of Single‐Cell Suspension

Single cell suspensions of mouse hippocampus tissue were prepared according to the protocol previously described [55]. Mice were deeply anesthetized with pentobarbital sodium and transcardially perfused with ice‐cold saline. The hippocampus was quickly extracted and washed in pre‐chilled HBSS. For the ChIP‐nexus experiment, hippocampus from five mice were pooled to form one replicate, with two replicates per group. The samples were then mechanically and enzymatically dissociated using the Neural Tissue Dissociation Kit (Miltenyi Biotec) following the manufacturer's protocol. After myelin removal with the Debris Removal Solution (Miltenyi Biotec), the cell pellet was resuspended in HBSS with 1% fetal bovine serum and used for formaldehyde crosslinking.

### 5.11 ChIP‐nexus

ChIP‐nexus experiments were conducted based on previous protocol with minor modifications [56]. Cells were cross‐linked with 1% formaldehyde for 10 min, followed by glycine quenching. The cells were collected by centrifugation and lysed twice with 1 mL of ChIP buffer (containing EDTA, TritonX‐100, Tris–HCl, NaCl, sodium deoxycholate, and protease inhibitors). After sonication for DNA fragmentation, the supernatant was obtained by centrifugation and incubated overnight at 4°C with rabbit anti‐PU.1 antibody (CST; 2258S). Magnetic protein A/G beads were used to enrich targeted chromatin, which was washed, and the DNA was eluted and purified for library preparation. DNA ends were blunted, dATP was added, and specific adaptors were ligated. The DNA was digested with exonucleases and reverse cross‐linked at 65°C overnight. DNA was extracted, precipitated with ethanol, and denatured to generate single‐stranded DNA (ssDNA). The ssDNA was circularized with ssDNA ligase and annealed with an oligonucleotide containing a *Bam*HI site. After *Bam*HI digestion and ethanol re‐precipitation, the DNA library was gel‐purified and sequenced on an Illumina platform.

### 5.12 Data Analysis

#### 5.12.1 Proteomic Data Analysis

DIA raw data were analyzed using Spectronaut software. For quantification, six peptides per protein and three daughter ions per peptide were chosen. The parameters included protein FDR ≤ 0.01, peptide FDR ≤ 0.01, peptide confidence ≥ 99%, and XIC width ≤ 75 ppm. Modified and shared peptides were excluded, and peak areas were summed for quantification. Proteins with at least one unique peptide were identified. The Majorbio Cloud platform was used for proteomic data analysis. *p* Values and fold changes (FCs) between sham and BCAS groups were calculated using the R package “*t*‐test.” To address the issue of proteins detected only in one group, we employed standard imputation methods allowing log2FC calculations [57, 58]. Proteins with significant differences (*p* < 0.05) were included in DEP analyses and functional studies. Considering potential visual artifacts, proteins exclusively expressed in one group were excluded from the volcano plot to ensure clarity and accurate interpretation. However, these proteins were included in downstream analyses, as they may provide biologically relevant insights. DEPs were defined by FCs (>2 or <0.5) and a *p* value < 0.05. Functional annotation was performed using GO.

#### 5.12.2 ATAC‐seq

Raw sequencing data were obtained in FASTQ format and processed with Fastp to filter low‐quality reads. Clean paired‐end data were aligned to the M. musculus reference genome (UCSC mm10) using Bowtie2. Picard tools were used to sort, index, and remove duplicates from the bam files. Peak calling was conducted using MACS2. Differential peaks were identified using the R package DiffBind, and peak annotation was done with the R package “ChIPseeker.” Motif analysis was conducted with HOMER, followed by TF annotation.

#### 5.12.3 Integrative Analysis

The integrated proteome and transcriptome analyses were conducted using the Majorbio Cloud platform (https://cloud.majorbio.com). For hippocampus‐specific integration of RNA‐seq (GSE223580) and ATAC‐seq (GSE246972) datasets, DAR‐related genes and DEGs were identified. Dot plots were generated with the R package ggplot2, and heatmaps were created using pheatmap. Venn diagrams from the VennDiagram package were used to find shared up‐ and downregulated genes in both datasets. Multiomics integration of transcriptome, epigenome, and proteome was performed to identify shared upregulated DAR‐related genes, DEGs, and DEPs. The shared 108 upregulated DEGs were identified using the Venn diagram tool (https://bioinformatics.psb.ugent.be/webtools/Venn/), and GO enrichment analysis was carried out using ShinyGO v0.77. Additionally, integrative analysis of hippocampus‐specific multiomics and published scRNA‐seq data was performed.

Cell clusters were created using Seurat and UMAP visualization based on the scRNA‐seq dataset (GSE60361) [22]. Enrichment analysis was conducted with shared upregulated DEGs. The *UCell* algorithm (https://github.com/chuiqin/irGSEA) was used to perform cell‐type deconvolution.

#### 5.12.4 Data Analysis of ChlP‐nexus

Raw reads were processed with Cutadapt (v2.10) to trim the first 10 bp barcode and adaptors. Reads longer than 10 bp posttrimming were aligned to the M. musculus reference genome (UCSC mm10) using Bowtie2 (v2.2.5). Samtools (v1.12) was used to merge, sort, and index reads from repeated samples. Narrow peaks were identified with MACS2 (v2.2.7.1) at a q‐value cutoff of 0.001, and overlapping peaks were selected using Bedtools (v2.30.0). Venn diagrams were created using the R package Vennerable. Mapped read coverage was calculated using Bedtools' genomecov function, and read normalization to RPKM was performed with Deeptools' bamCoverage module (v3.5.1). Heatmaps were generated with Deeptools' plotHeatmap module. Differential peaks (FDR < 0.05) were identified with DiffBind, and peak annotation was done using ChIPseeker. ChIP‐nexus signals were visualized with IGV, and GO analysis of biological processes was performed with ClusterProfiler.

### 5.13 Statistical Analysis

All experiments and data analyses were conducted with blinding to group assignments. Statistical analyses were performed using GraphPad Prism. Paired *t*‐tests (two‐tailed) compared the 0.18 and 0.16 mm sides within the BCAS group, while unpaired *t*‐tests (two‐tailed) compared sham and BCAS groups. One‐way and two‐way ANOVA were used for multiple group comparisons. Data are presented as mean ± SEM, with significance set at *p* < 0.05.

## Author Contributions

The study was conceived by Jing Zhao. Zengyu Zhang and Dewen Ru were responsible for designing and executing the experiments, analyzing the data, and drafting the manuscript. Zhuohang Liu and Yong Wang provided assistance with the experimental procedures and contributed to the data analysis. The ChIP experiments were conducted with the help of Yong Wang, Zimin Guo, and Lei Zhu. Yuan Zhang and Min Chu carried out the analysis of the ATAC‐seq data. Jing Zhao and Yong Wang provided overall study conceptualization, experimental supervision, and manuscript revision. All authors have reviewed and approved the final version of the manuscript.

## Ethics Statement

This study adhered to the ethical guidelines of Fudan University's Animal Care and Use Committee. Ethical approval was granted by the Department of Laboratory Animal Science, Fudan University (Approval Number: 2023‐MHFY‐23JZS).

## Conflicts of Interest

The authors declare no conflicts of interest.

## Supporting information



Supporting Information

Supporting Information

Supporting Information

Supporting Information

Supporting Information

Supporting Information

Supporting Information

Supporting Information

## Data Availability

The datasets generated during this study are available in the online repository, with the repository name(s) and accession number(s) provided below: https://www.ncbi.nlm.nih.gov/geo/query/acc.cgi?acc=GSE223580. https://www.ncbi.nlm.nih.gov/geo/query/acc.cgi?acc=GSE246972.
