# Supplementary material for: Integrative Multiomics Profiling of Mouse Hippocampus Reveals Transcriptional Upregulation of Interferon‐Stimulated Genes Through PU.1 Regulator in Microglial Activation Induced by Chronic Cerebral Hypoperfusion
Source: MedComm (2020). 2025 Apr 15;6(5):e70157. doi: 10.1002/mco2.70157 (PMC11999893; doi:10.1002/mco2.70157)
Supplement: Supplementary file 1 — Supporting Information [file MCO2-6-e70157-s005.pdf]

Integrative multi-omics profiling of mouse hippocampus reveals transcriptional upregulation of interferon-stimulated genes through PU.1 regulator in microglial activation induced by chronic cerebral hypoperfusion

Zengyu Zhang[1,3#], Dewen Ru[2,3,4#], Zhuohang Liu[1,3], Zimin Guo[5], Lei Zhu[6], Yuan Zhang[7], Min Chu[1], Yong Wang[8*], Jing Zhao[1,9*]

[1]Department of Neurology, Minhang Hospital, Fudan University, Shanghai 201199, China
[2]Department of Neurosurgery, Jinshan Hospital, Fudan University, Shanghai 201508, China
[3]Shanghai Medical College, Fudan University, Shanghai 200032, China
[4]Department of Neurosurgery, Huashan Hospital, Fudan University, Shanghai 200040, China
[5]Department of Neurology, Shanghai Pudong Hospital, Fudan University Pudong Medical Center, Shanghai 201399, China
[6]Department of Vascular Surgery, Huashan Hospital, Fudan University, Shanghai 200040, China
[7]Department of Vascular Surgery, Shanghai Pudong Hospital, Fudan University Pudong Medical Center, Shanghai 201399, China
[8]Department of Neurology, Zhongshan Hospital, Fudan University, Shanghai 200030, China
[9]Institute of Healthy Yangtze River Delta, Shanghai Jiao Tong University, Shanghai 200001, China

*Correspondence author:
Jing Zhao, Department of Neurology, Minhang Hospital, Fudan University, Shanghai 201199, China. E-mail address: zhao_jing@fudan.edu.cn;
Yong Wang, Department of Neurology, Zhongshan Hospital, Fudan University, Shanghai 200030, China. E-mail address: yong_wang@fudan.edu.cn

#Zengyu Zhang and Dewen Ru contributed equally to this study.

Figure S1. Hippocampal neuropathological changes induced by hypoperfusion following BCAS. (A) Representative Nissl-stained images showing morphological changes in the hippocampus. (B) Quantification of neuronal density in the CA1 region based on Nissl staining. One-way ANOVA. (C) Exemplary HE staining of hippocampal sections. (D) Iba-1-stained coronal sections from sham and BCAS mice, illustrating microglial activation. (E) Comparison of relative microglial activation intensity between 0.18 mm and 0.16 mm sides in BCAS mice. (F-H) Immunofluorescence staining of Iba-1/NeuN in CA1 and CA3 hippocampal regions from both sham and BCAS groups. N = 8 mice per group. Data are presented as mean ± SEM. NS = not significant, ***$p < 0.001$.

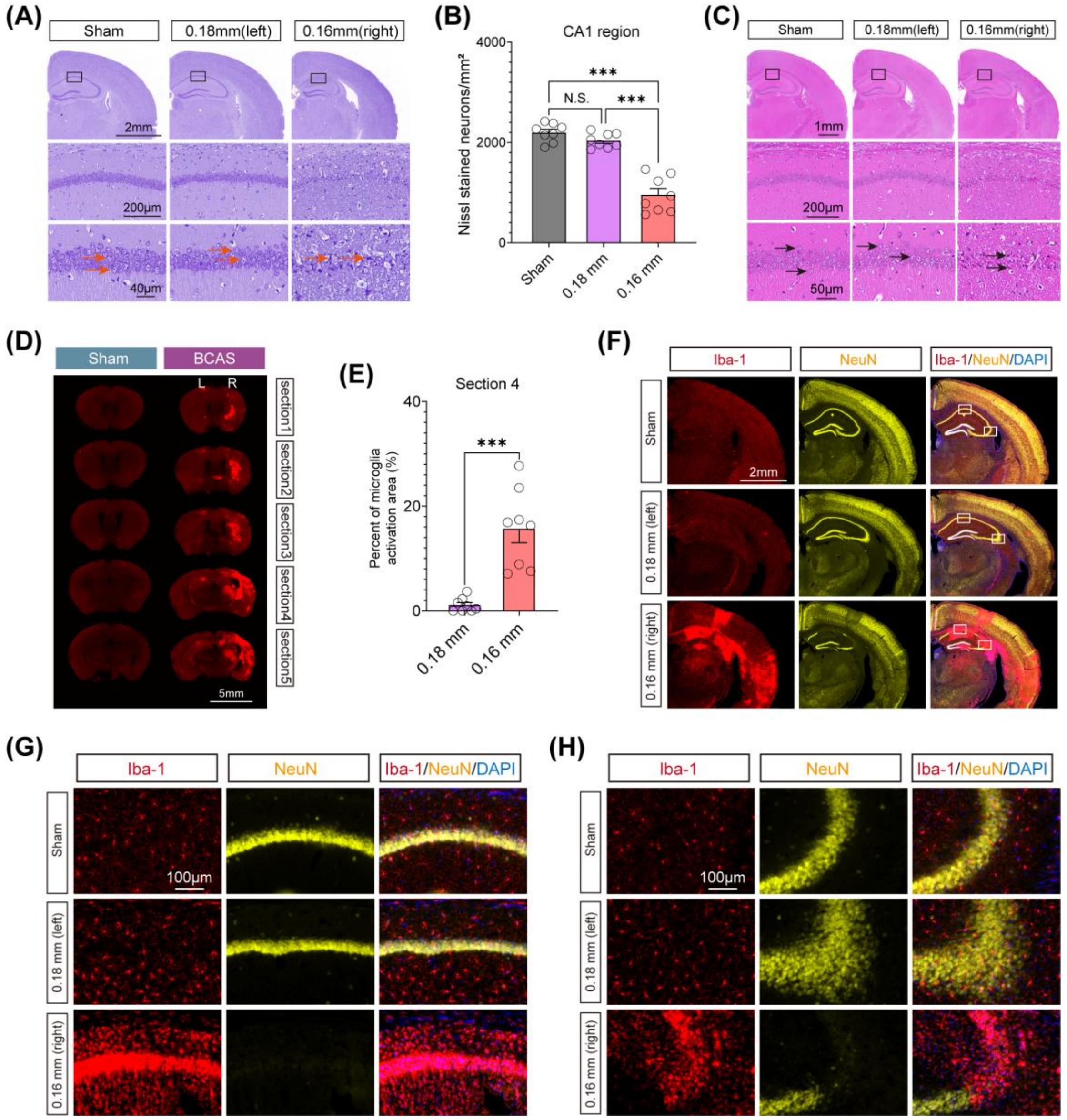

Figure S2. The quality control of mass spectrometry data. (A) Protein molecular weight distribution. (B) Peptide number distribution. (C) Peptide length distribution. (D) Distribution of Protein's Sequences Coverage. (E) CV distribution between sham and BCAS group.

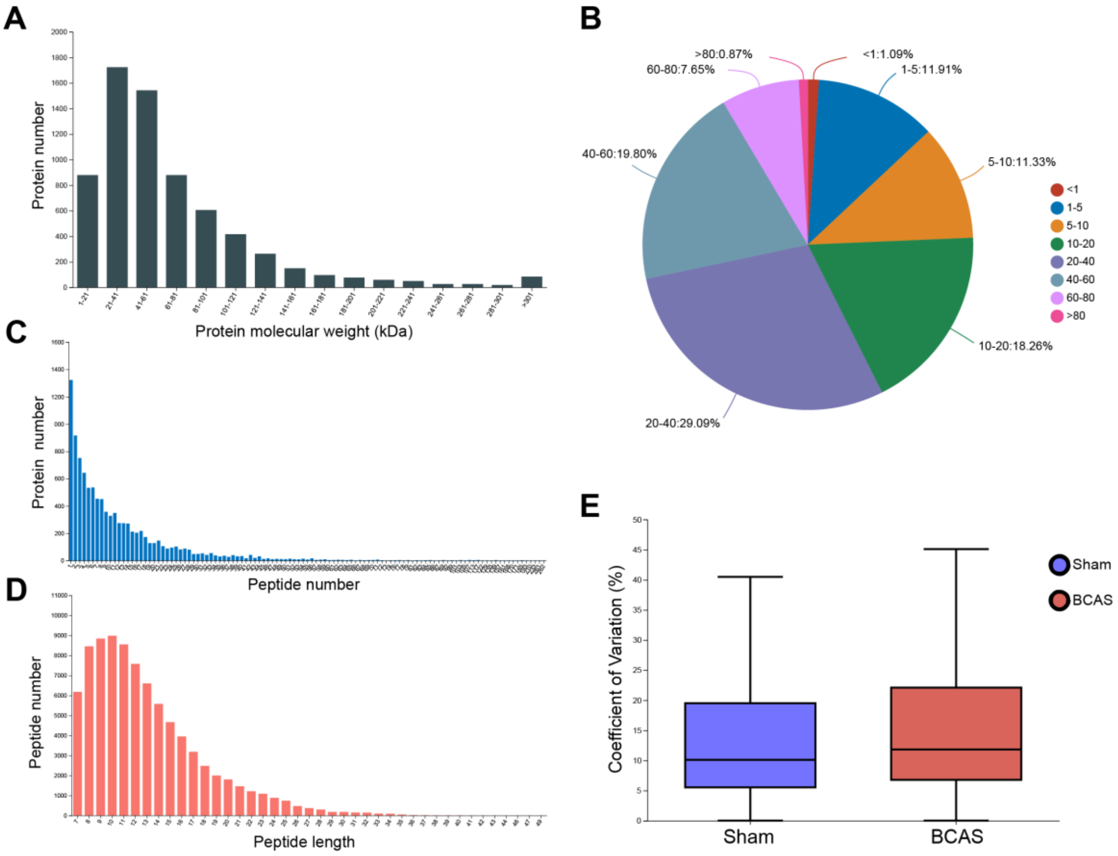

Figure S3. Proteomics analysis revealed differentially expressed proteins (DEPs) in hippocampal tissue following BCAS-induced hypoperfusion. (A) Venn diagram showing overlapping proteins between sham and BCAS groups. (B) Heatmap of correlation analysis between the two groups (N = 4 biological replicates per group). (C) Volcano plot displaying DEPs in the hippocampus post-BCAS. (D) Heatmap clustered by both samples and protein categories (N = 4 per group). (E) GO enrichment analysis of all DEPs between the two groups. (F-G) Bar plots illustrating enriched GO biological processes for upregulated and downregulated proteins.

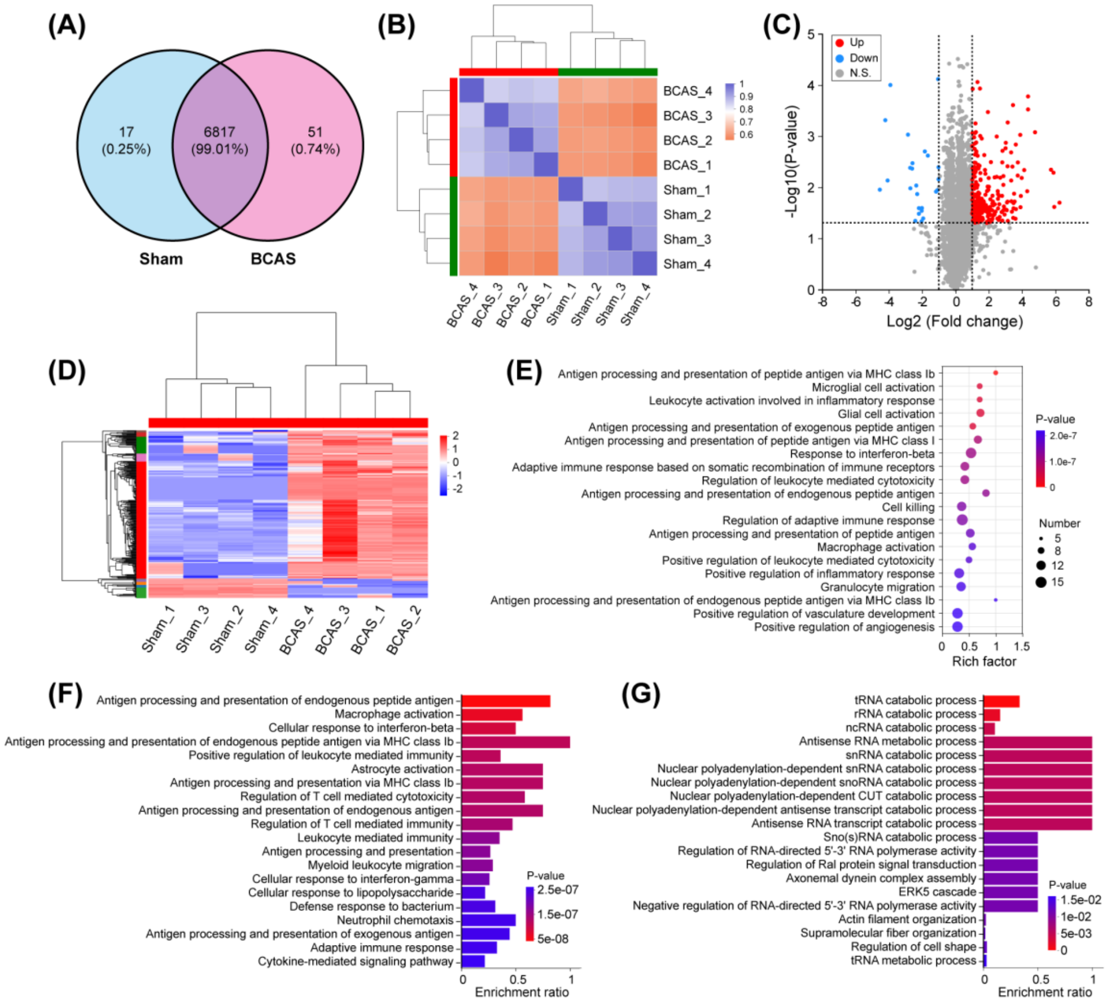

Figure S4. Verification of differential up-regulated molecular expression at transcriptional and protein levels. (A) qRT-PCR was utilized to validate the differential gene expressions on isolated hippocampus from sham- and BCAS-operated mice. mRNA levels were quantified relative to the housekeeping gene *Gapdh*. Up-regulated genes involved in “Response to interferon-beta pathway” *(Gbp2*, *Irgm1*, *Ifitm3*, *Gbp7*, *Bst2*)” and “Microglial cell activation pathway” (*Trem2*, *Tlr2*, *Tlr3*, *Aif1*, *Grn*, *C1qa, C5ar1*). Data are presented as mean ± SEM. Unpaired two-tailed t-test was used for statistical analysis, *$p < 0.05$, **$p < 0.01$, ***$p < 0.001$. N = 6 mice per group. (B) Representative confocal images of Iba-1/GBP2 staining. NC represents a negative control without the GBP2 primary antibody, showing only secondary antibody staining. (C) High-resolution confocal images of Iba-1/GBP2 immunofluorescence highlighting GBP2-positive microglial cells (arrowheads) in BCAS mice. NC confirms that the staining is not due to secondary antibody background.

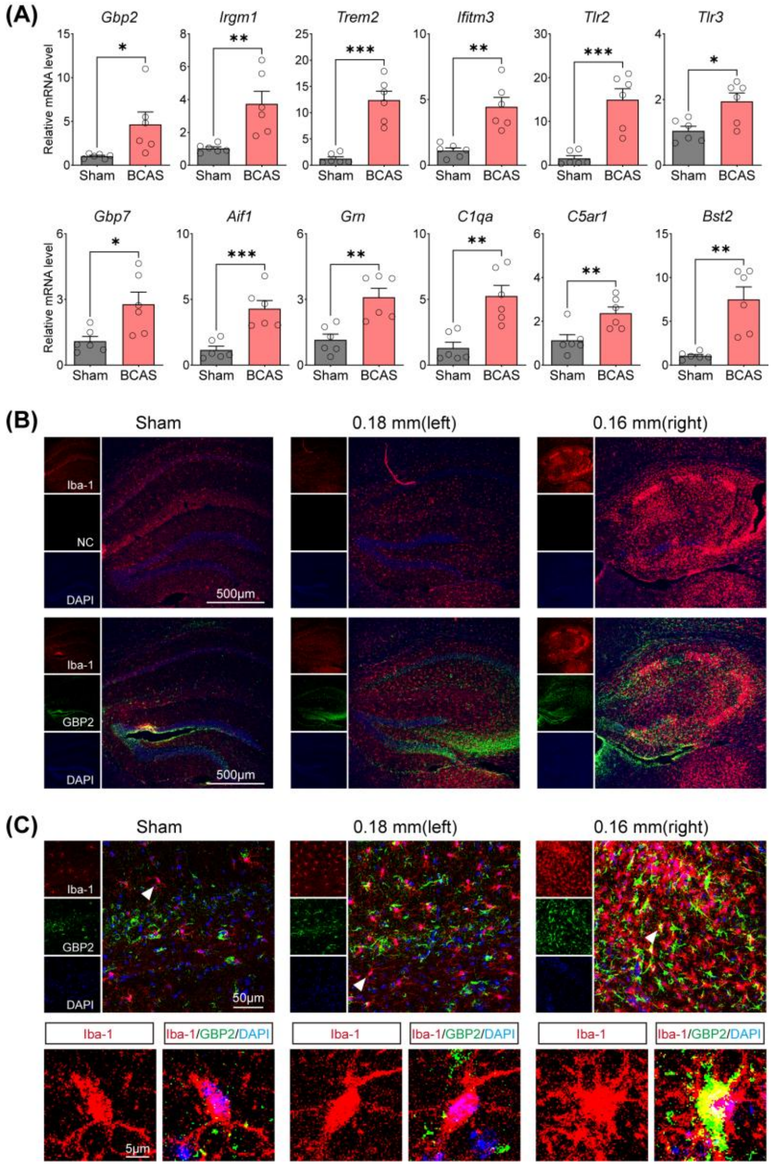

Figure S5. Immunofluorescence staining demonstrated microglial activation in the hippocampus of BCAS mice. (A) Representative high-resolution images of brain slices labeled with Iba1, showing microglial activation (arrowheads) in BCAS mice (60x). No GBP2 primary antibodies were used in these experiments, ruling out background signals from the secondary antibody, as evidenced by the negative control. (B) Quantification of GBP2-positive cells in both sham and BCAS groups. Data are presented as mean ± SEM. One-way ANOVA test, NS = not significant, ***$p < 0.001$.

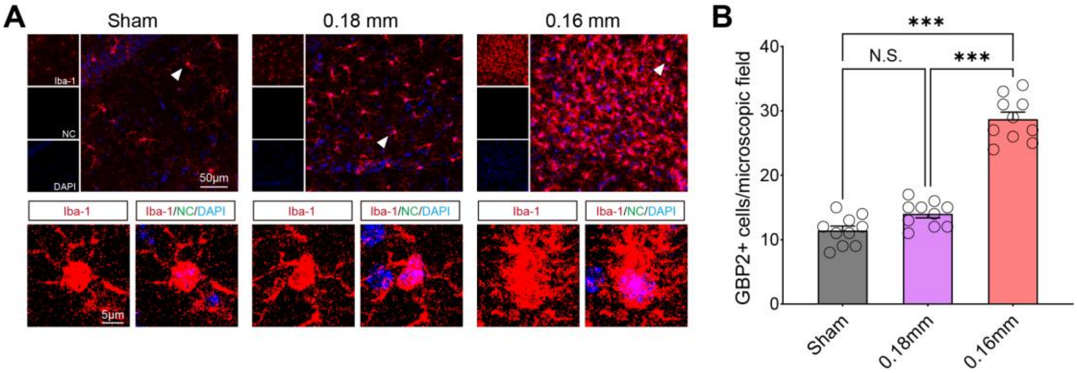

Figure S6. The ATAC-seq data were evaluated for base content distribution. (A-D) Base content distribution of ATAC-seq reads in the sham group. (E-H) Base content distribution of ATAC-seq reads in the BCAS group.

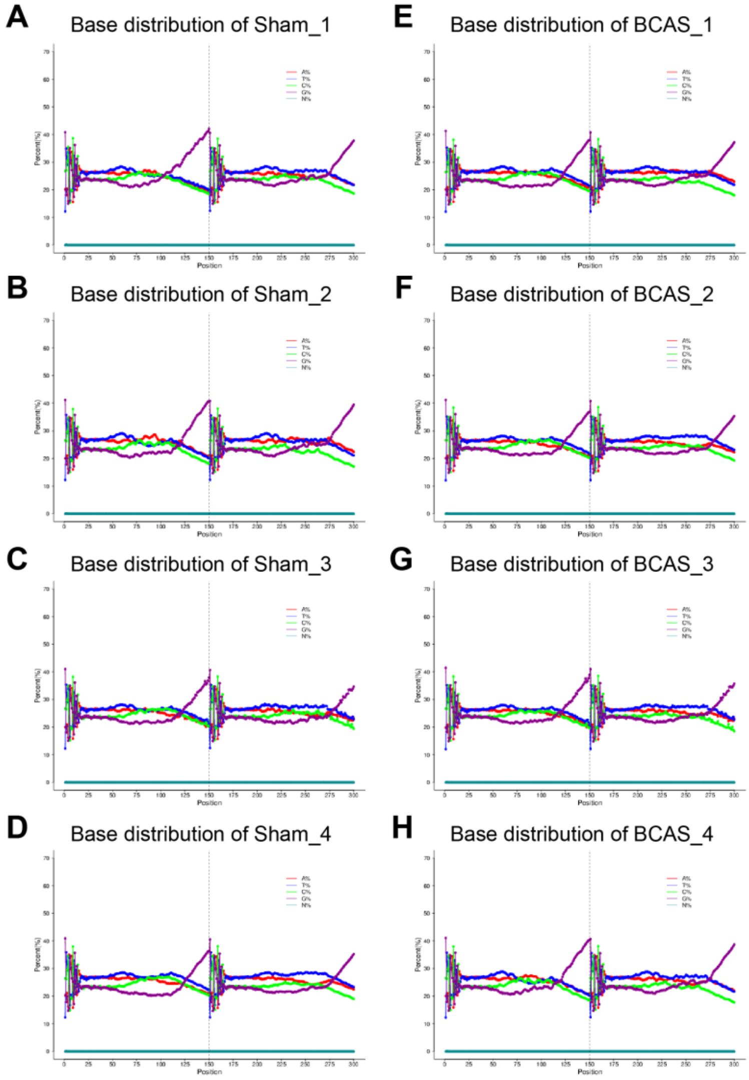

Figure S7. The ATAC-seq data were evaluated for distribution of mean sequence quality. (A-D) Mean sequence quality distribution of ATAC-seq reads in the sham group. (E-H) Mean sequence quality distribution of ATAC-seq reads in the BCAS group.

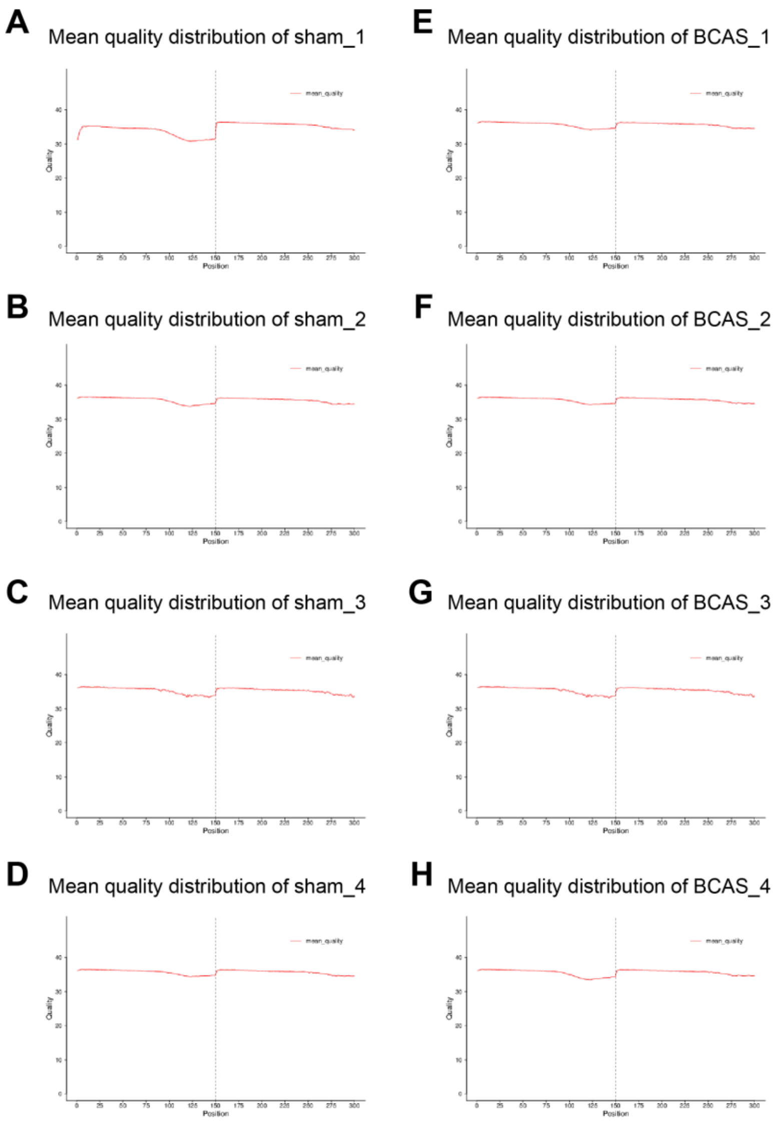

Figure S8. The characteristic phenotype of the activated microglia/macrophage was assessed using dual Iba1/CD68 immunostaining after BCAS-induced hypoperfusion. (A) Representative confocal images labeled with Iba1 and CD68. (B-C) High-resolution images of Iba1/CD68 immunofluorescence revealed microglia positive for CD68 (indicated by arrowheads) in BCAS mice. Note that the activated microglia underwent significant morphological changes, transitioning to an amoeboid-like phenotype. This transformation is characterized by features typically associated with phagocytic activity. (D-E) Quantitative analysis of proportion of Iba1+ cells that are CD68+, and the number of Iba1+ microglia. One-way ANOVA test. N=13 microscopic field per group. Data are expressed as mean ± SEM. NS = not significant, ****p* < 0.001.

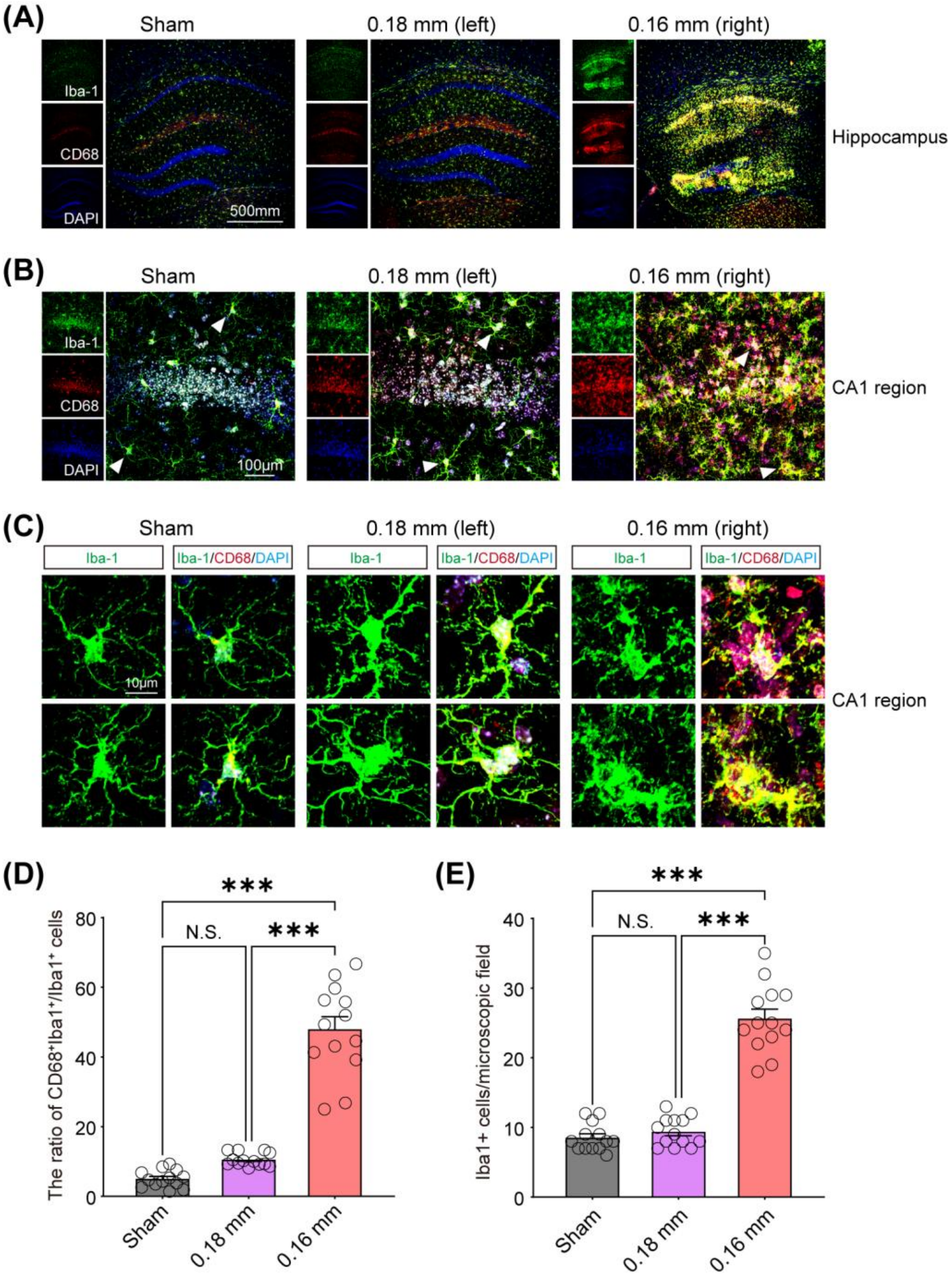

Figure S9. Genome browser tracks of ChIP-nexus signals at representative target gene loci. Black rectangles highlight the up-peak regions of PU.1 on target gene promoters. Notably, the promoter regions (≤ ±3 kb to TSS) of *Tlr2*, *Ccl7* and *Ccl12* were significantly influenced by PU.1, suggesting that PU.1 may enhance the transcription of these genes.

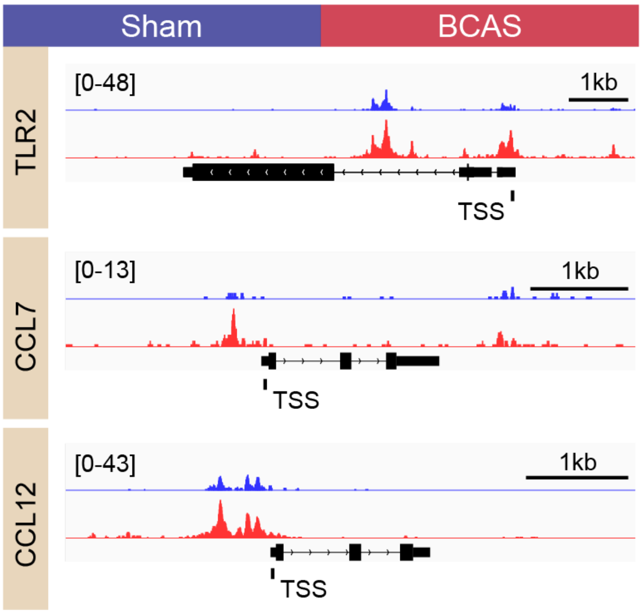

Figure S10. Downstream analysis was confirmed through Iba-1/STING double-staining following BCAS-induced hypoperfusion. Representative immunofluorescence staining images labeled with Iba-1 and STING in the hippocampus of both sham and BCAS groups.

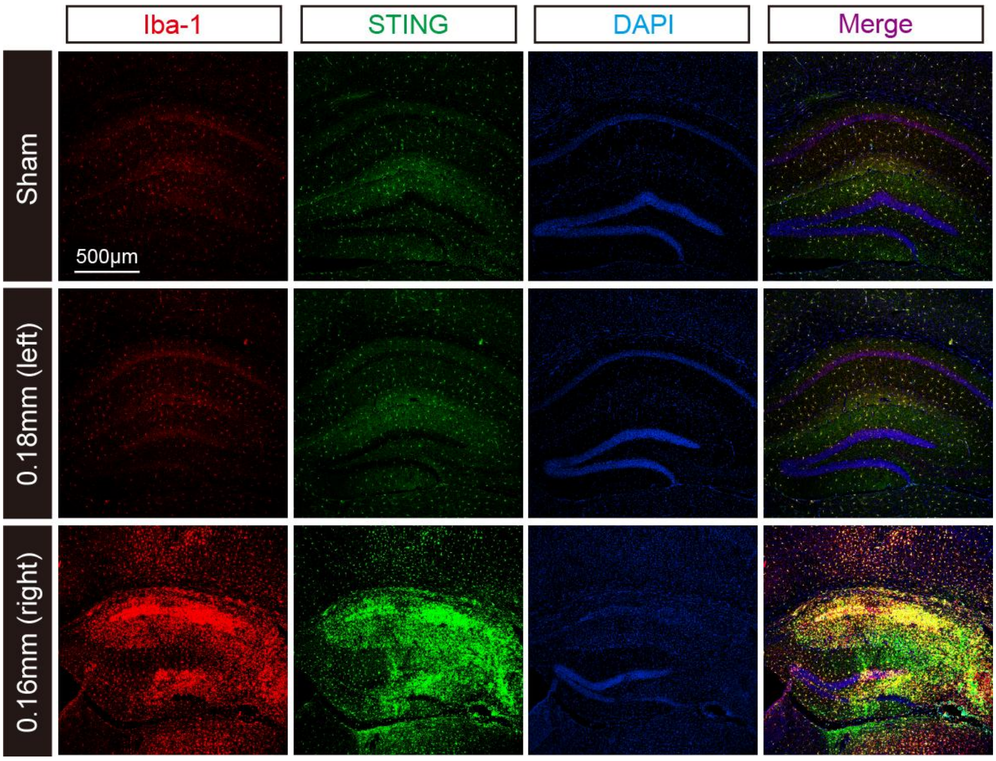

Table S1. Primer sequences used for qRT-PCR.

Table S2. DEPs obtained by proteome data.

Table S3. Shared DEGs obtained by transcriptome and proteome data.

Table S4. DARs obtained by ATAC-seq.

Table S5. Upregulated DAR-related genes, DEGs and DEPs.

Table S6. Downregulated DAR-related genes and DEGs.

Table S7. Shared upregulated DAR-related genes, DEGs and DEPs.
